# Identifying models of dielectric breakdown strength from high-throughput data via genetic programming

**DOI:** 10.1038/s41598-017-17535-3

**Published:** 2017-12-14

**Authors:** Fenglin Yuan, Tim Mueller

**Affiliations:** Department of Materials Science and Engineering, Johns Hopkins University, Baltimore, MD 21218 USA

## Abstract

The identification of models capable of rapidly predicting material properties enables rapid screening of large numbers of materials and facilitates the design of new materials. One of the leading challenges for computational researchers is determining the best ways to analyze large material data sets to identify models that can rapidly predict a given property. In this paper, we demonstrate the use of genetic programming to generate simple models of dielectric breakdown based on 82 representative dielectric materials. We identified the band gap E_g_ and phonon cut-off frequency ω_max_ as the two most relevant features, and new classes of models featuring functions of E_g_ and ω_max_ were uncovered. The genetic programming approach was found to outperform other approaches for generating models, and we discuss some of the advantages of this approach.

## Introduction

With the ever-increasing power of supercomputers, materials scientists are able to perform high-throughput density functional theory (DFT) calculations^1,2^ and build up online databases^3–6^ of important materials properties including structure parameters, thermodynamic and transport properties, and electronic structures and properties. Such vast amounts of materials information enables the use of machine learning methods to identify simple predictive models of more complex material properties^7–18^. Such simple models can be used to rapidly screen materials, enabling experimentalists focus on only the most promising candidates and expediting the development of new materials with a reduced cost.

There are two challenges in this approach to materials discovery and design: it is necessary to generate well-organized and high-quality data, and it is necessary to use suitable machine learning algorithms to identify the most relevant models. To address the first challenge, there are several active projects to create broadly accessible databases of high-quality material data^3–6^. The best approach to deal with the second challenge is not clear, as there are a wide variety of machine learning algorithms that could be used and different algorithms are appropriate for different problems^11^. In this paper, we demonstrate the power of a supervised learning algorithm known as “genetic programming”, in which an evolutionary algorithm is used to perform symbolic regression, for identifying simple models for dielectric breakdown strength of materials. Mueller *et al*.^19^ have previously demonstrated that genetic programming can be used to identify important structural descriptors for hole trap depths in hydrogenated nanocrystalline and amorphous silicon, and here we evaluate the genetic programming approach for the identification of predictive models of dielectric breakdown strength.

## Methods

### Genetic programming

Genetic programming (GP) is a supervised machine-learning algorithm in which the hypothesis space (the space of functions to be considered) consists of combinations of simple functions and operators (e.g., addition, subtraction, exponentiation, square root, etc.). The goal of the genetic programming algorithm, as in any supervised learning algorithm, is to find a function within this hypothesis space that is able to best predict the output value of interest, which in our case is the calculated dielectric breakdown strength. To search this hypothesis space a genetic or evolutionary algorithm is used to evolve a population of candidate functions (or models) according to natural-selection rules, in which good models are retained, bad models are tossed out, and new models are created by crossover (combining existing models) and mutation (modifying existing models). The fittest models should achieve a balance between complexity, speed and accuracy. This approach has been widely applied in multidisciplinary fields, including but not limited to financial market analysis^20^, biological science^21,22^, software development^23^, and identifying interatomic potential models from calculated energies^24–26^. In materials science and engineering, researchers have used genetic programming to develop predictive models for properties of concrete and cement^27–29^, asphalt^30^, shape memory alloys^31^, and heterogeneous catalysts^32^. They have also been used to determine the effects of processing parameters on metal alloys^33–36^, predict the impact roughness of cold formed materials^37^, optimize productivity for the steel industry^38^, and develop models for a variety of problems in structural engineering^39^. Recently genetic programming has been identified as a useful tool for extracting important descriptors of material properties from computational data^19^.

### Test and training data

Here we evaluate the effectiveness of genetic programming in predicting dielectric breakdown strength. Dielectric breakdown strength is defined as the maximum external electric field strength that the materials can withstand before turning into a conductor. Materials with high dielectric breakdown strength are used as insulators for applications including high voltage power transmission and capacitors^40–42^. Fundamentally, dielectric breakdown strength is a complex phenomenon involving physical interaction between materials and an electric field. Here, we focus on intrinsic dielectric breakdown strength, which is defined for a defect-free crystal and theoretically is only influenced by materials chemistry. The calculation of intrinsic dielectric breakdown strength is based on von Hippel^43^ and Fröhlich^44–46^ theory implemented in a DFT framework^47^. Due to the time-consuming nature of such calculations, only 82 representative crystals were calculated. The details of these calculations and the underlying theories can be found in the work by Sun *et al.* ^47^ and Kim *et al.* ^48^.

Despite its importance in both academia and industry, dielectric breakdown strength lacks a good predictive model that can be used to rapidly screen new candidate materials. Recently Kim *et al*.^48^ provided a case study of searching for relevant models via three supervised machine-learning algorithms: Kernel Ridge Regression (KRR)^49–51^, Random Forest Regression (RFR)^49^ and Least Absolute Shrinkage and Selection Operator (LASSO)^49,52^. Of these, they determined that the LASSO method was effective for the identification of analytical models, and based on this method they developed several phenomenological models for crystalline dielectric materials. They highlight the following model as being particularly good in terms of simplicity and accuracy:

1 $${F}_{b}=24.442{e}^{0.315\sqrt{{E}_{g}{\omega }_{\max }}},$$

or, equivalently

2 $$\mathrm{ln}({F}_{b})=3.196+0.315\sqrt{{E}_{g}{\omega }_{\max }},$$

where F_b_ is the dielectric breakdown strength, *E*_*g*_ is the electronic band gap, and *ω*_max_ is the maximum phonon frequency.

The genetic programming method used in this paper is in some ways similar to the LASSO method. The LASSO method used by Kim *et al.* ^48^ assesses linear combinations of elementary terms, where each term is a function of some subset of material properties. The disadvantage to this approach is that a list of possible terms must be provided to the algorithm. (Kim *et al.* ^48^ combinatorially generated a total of 187,944 terms, each containing functions of up to three properties.). In contrast, genetic programming is capable of dynamically generating such terms by combining properties in non-linear ways, so only the list of known material properties must be provided to the algorithm.

To enable comparison with LASSO and other machine learning algorithms, we have applied the genetic programming approach to the same input dataset generated by Kim *et al*.^48^. This data set is composed of eight feature properties, listed in Table 1, for 82 representative crystalline dielectric materials. The detailed information for 82 crystalline insulators can be found in the supplementary information of Kim *et al*.’s paper^48^.

**Table 1:** Eight feature properties related to dielectric breakdown strength.

Name	Symbol	Name	Symbol
Band Gap	*E*_*g*_	Dieletric constant	*d*_*t*_
Phonon frequency (max)	*ω*_max_	Dielectric constant (electron)	*d*_*e*_
Phonon frequency (mean)	*ω*_mean_	Nearest Neighbor Distance	*a*
Density	*ρ*	Bulk Modulus	*bm*

### Simulation Details

We performed genetic programming calculations using the Eureqa software package^53^. In genetic programming, an explicit definition of elementary operators is required to define the hypothesis space, and in this study we chose a collection of four algebraic operators (i.e., plus, minus, division, multiplication) and three function operators (square root, exponential and logarithm functions). For each of these operators we used the default complexity value in Eureqa (see Supplementary Table S1). We used three different but representative metrics to measure the fitness of candidate models: the mean absolute error (MAE), the root mean square error (RMSE) and the Pearson correlation coefficient (PCC) (as defined in the SI). The PCC assesses the degree to which two sets of data are linearly related, regardless of how close they are to each other in value. Thus when the PCC is used as the objective function, the breakdown strengths predicted by the models output by Eureqa need to undergo a linear transformation to enable direct comparisons with the DFT-calculated breakdown strengths (see Supplementary Fig. S1). On the other hand the use of MAE or RMSE as the objective function produces output that is directly comparable to the training data. Although this obviates the need for a linear transformation of the model output, it imposes an additional requirement on the model output that could make the discovery of simple, accurate models more difficult.

To estimate the predictive performance of the evaluated models, we set the parameters in Eureqa so that in each run the 82 materials in the data set were randomly partitioned into two groups of the same size (i.e., each containing 41 materials): the training and the validation dataset. Model optimization is done using the training set, and the validation set is used to construct a Pareto frontier of models, defined as the set of models for which no other models were both simpler and more accurate. We take the output of a single Eureqa run to be the set of models on the Pareto frontier after total number of evaluated models reached 10^12^.

## Results and Discussion

### Evaluation of individual properties and products of properties

To estimate the degree to which each of the eight feature properties is related to the dielectric breakdown strength, we first simply count the number of times each appears in a model found by Eureqa. Because genetic programming is a stochastic method, with randomness in both the evolutionary algorithm and the way in which the validation set is selected, we performed our analysis over eight independent runs for each objective function. We define a parameter f_i_ by:

3 $${f}_{i}={N}_{i}/N,$$

where N is the total number of models in the Pareto frontier and N_i_ is the total number of models in the Pareto frontier that are functions of the the i^th^ material property. Higher f_i_ values for a particular property suggest that the property is more useful as a descriptor of dielectric breakdown strength.

**Figure 1:**
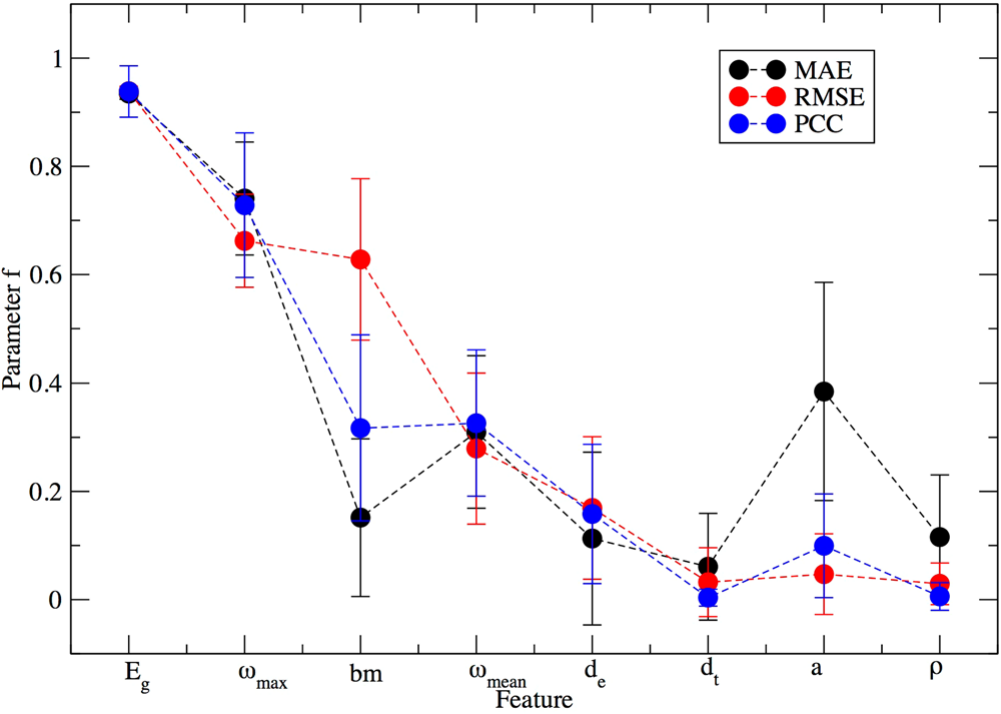
The frequency with which each of the eight features appears on the Pareto frontier (parameter f). Error bars are from eight parallel Eureqa runs.

The calculated values of f_i_ for the eight material properties are shown in Fig. 1. The properties E_g_ (the band gap) and ω_max_ (the maximum phonon frequency) have the highest values of f_i_. For all three objective functions, more than 60% of the models on the Pareto frontier contain these values. The importance of E_g_ and ω_max_ in predicting dielectric breakdown strength is in agreement with the results obtained by the machine learning models evaluated by Kim *et al*.^48^, as well as a simple correlation analysis (Supplementary Fig. S3). These results indicate that genetic programming is an effective tool for rapidly identifying the most relevant properties, consistent with prior results^19^. Our results also revealed that bm (the bulk modulus) and ω_mean_ (the average phonon frequency) have the next-highest values of f_i_, which is not surprising given the degree to which these properties are correlated with ω_max_ (see Supplementary Fig. S3). These three properties (ω_max_, ω_mean_, and bm) are associated with the stiffness of the material^54^, which is consistent with the proposed physical picture described by Kim *et al*.^48^.

**Figure 2:**
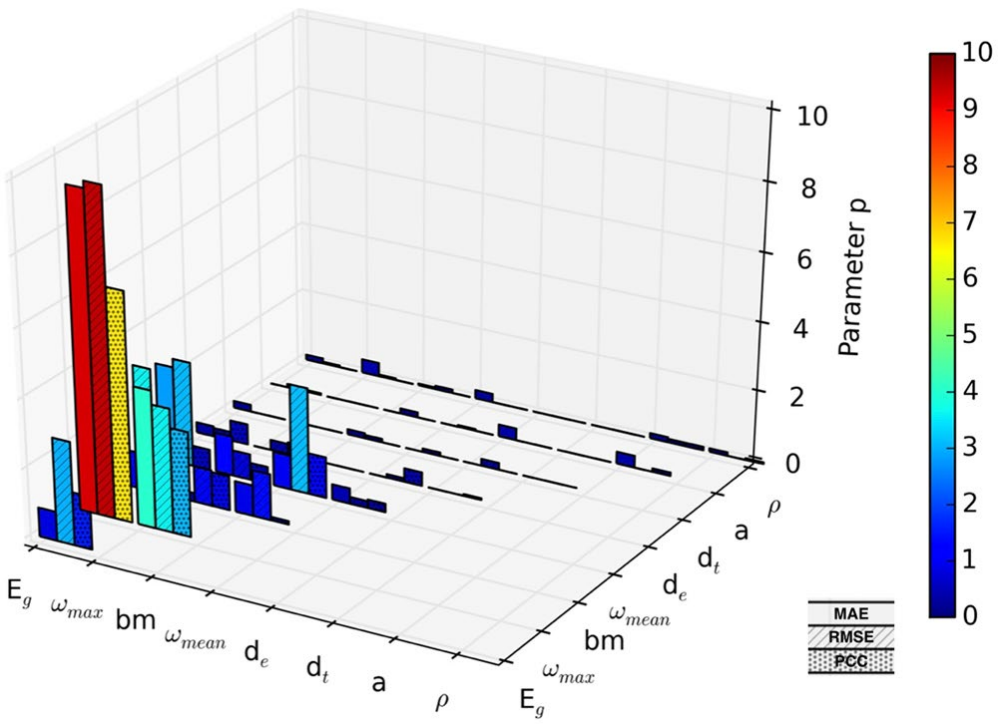
Distribution of the frequency of occurrence (parameter p) of products of two features.

After scrutinizing the raw results from Eureqa, it was evident that the multiplication of two features is the most frequent way for features to be combined. Based on this observation, we computed the number of appearances for all possible products of two features (Fig. 2). For all three objective functions the E_g_*ω_max_ term appeared most frequently, providing further evidence of the importance of these two properties.

### Performance of the genetic programming models

We first consider a direct comparison between the results of genetic programming and the results obtained by Kim *et al*.^48^. To make this comparison, we have used the logarithm of the dielectric breakdown strength as the output value, to be consistent with their approach^48^. To accelerate the search, we restricted the properties considered to the two that consistently appeared most frequently in models on the Pareto frontier: E_g_ and ω_max_, based on Figs 1 and 2. All results in this paper are under this premise unless stated otherwise. Because the two-feature Eureqa runs are faster than the eight-feature runs, for each objective function we gathered statistics for 16 two-feature Eureqa runs. In each of these runs it took approximately 14 hours to evaluate 10^12^ models on a single core of 3 GHz Intel Core i5-2320 CPU.

**Figure 3:**
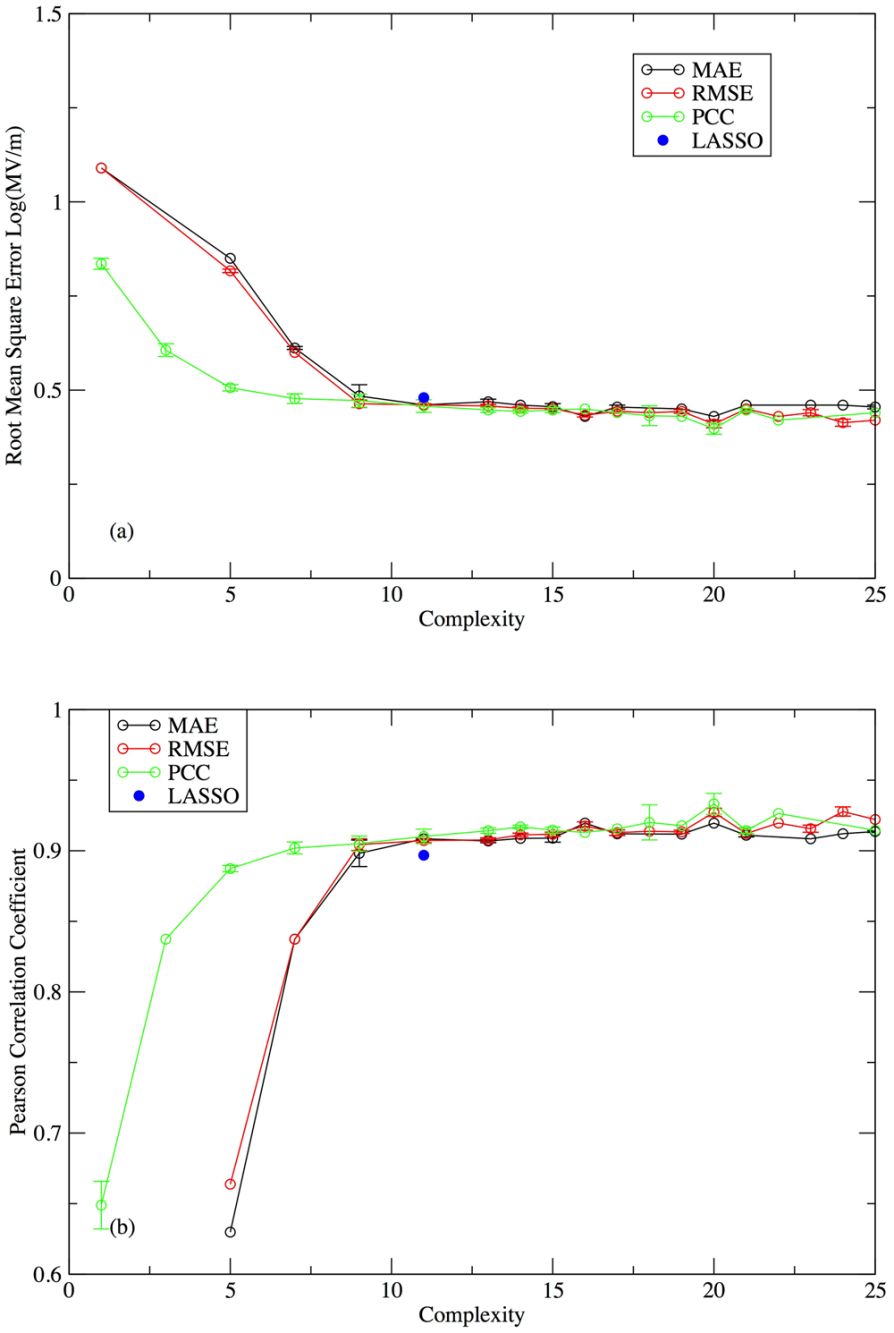
Average (**a**) RMSE and (**b**) PCC performance of models on total input data (training plus validation datasets) by MAE, RMSE, PCC optimizations compared with the LASSO solution. The error bar is the calculated standard deviation from averaging over 16 parallel Eureqa runs.

Averaged over all 16 Eureqa runs as a function of complexity, the performance of the generated models for all three objective functions (MAE, PCC, RMSE) on the total set of input data (i.e., training plus validation sets) increases with increasing complexity and levels off when the complexity reaches around 10 (Fig. 3). The LASSO solution (equation (2)) has a complexity of 11, determined via the same complexity measure used for the genetic programming runs. At this level of complexity, the average model found by genetic programming has slightly better performance than the LASSO solution on the training and validation data.

**Figure 4:**
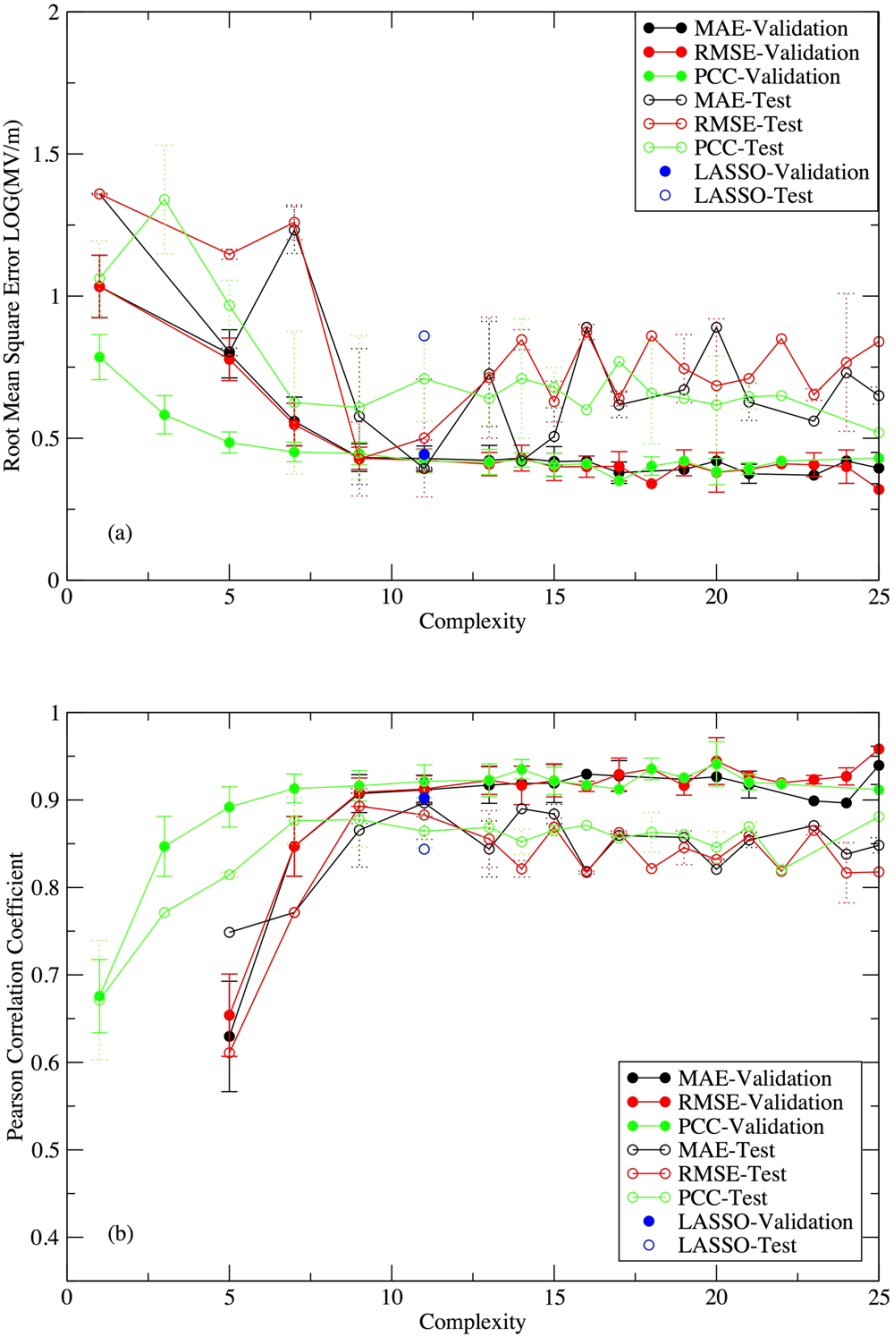
Average (**a**) RMSE and (**b**) PCC performance of models on validation and test sets by MAE, RMSE, PCC optimizations compared with the LASSO solution. The error bar is the calculated standard deviation from averaging over 16 parallel Eureqa runs.

To assess the predictive ability of the generated models, i.e. how well the models on the Pareto frontier perform when exposed to data Eureqa has never seen, we evaluated the models’ performance (measured by RMSE and PCC) on an out-of-range test set (Fig. 4). The test set consisted of four cubic crystals^48^, Li_2_S, Na_2_S, SrCl_2_ and ZrO_2_, as well as six perovskite crystals^55^, BaSnO_3_, CaGeO_3_, CaSiO_3_, BSiO_2_F, BaBO_2_F and SrBO_2_F. Detailed information about these ten materials is provided in the works by Kim *et al.* ^48,55^ and summarized in Supplementary Table S2. The test data was not used by either the genetic programming algorithm or the LASSO algorithm when generating models.

For the models discovered by genetic programming, the average quality of the predictions on the test data improves until about a complexity of about 10, at which point the errors start to increase (Fig. 4). In contrast, the performance of models on the validation data increases with increasing complexity and levels off when the complexity reaches about 10 (Fig. 4). These results indicate that beyond this complexity, the genetic programming algorithm is overfitting the data. Although the LASSO solution has performance similar to the genetic programming models on the validation data, its performance on test data is significantly worse, manifested by higher RMSE and lower PCC values. Its performance on the test data is comparable to the genetic programming models at levels of complexity that overfit the training data, suggesting that the LASSO solution may have also overfit the data. We note that the genetic programming algorithm found the LASSO solution in two of the 48 Eureqa runs, and in both of these runs PCC was the objective function. The PCC runs appear to be better than the RMSE or MAE runs at identifying models with good predictive ability at low levels of complexity, which is understandable given the additional requirements imposed on the generated models when MAE or RMSE is used as an objective function.

Our results suggest that genetic programming is effective at finding models with good predictive ability, provided that the appropriate level of complexity is determined. Models that are too simple are not able to adequately account for the factors that influence dielectric breakdown strength, and models that are too complex overfit the data and have relatively poor predictive ability. The challenge is in determining the appropriate level of complexity to minimize prediction errors. In addition, at a given level of complexity there may be many different models by different genetic programming runs, and it is also necessary to select from these models. One approach to identifying models that are expected to have good predictive accuracy is to simply withhold a set of test data, as we have done here. Approaches in which a set of test data is withheld have the added benefit of allowing for the amount of uncertainty in the predictions for each model to be estimated by evaluating the prediction errors on the withheld data^11^. Similarly, cross-validation could be used to try to identify the optimal level of complexity. Some amount of cross-validation is already included in the genetic programming algorithm, as the data is randomly partitioned into training and validation sets for each Eureqa run. Here we explore two alternative strategies for identifying the best models.

The first strategy we explored is to simply count the number of times a model appears in the different stochastic runs, under the hypothesis that models that appear on the Pareto frontiers more frequently are less likely to have fit the training data well by chance. In each of the 48 Eureqa runs, a different, randomly-selected partition of training and validation data was used. We counted the number of times each model appeared on the 48 Pareto frontiers, considering only the functional relationships between the two feature properties and ignoring differences in the constants (e.g. coefficients), as we found that for the same model the constants identified by Eureqa could differ slightly from run to run. We chose a single set of parameters to plot by using a gradient descent algorithm to identify the locally optimal parameters. (Details are provided in the supporting information.)

**Figure 5:**
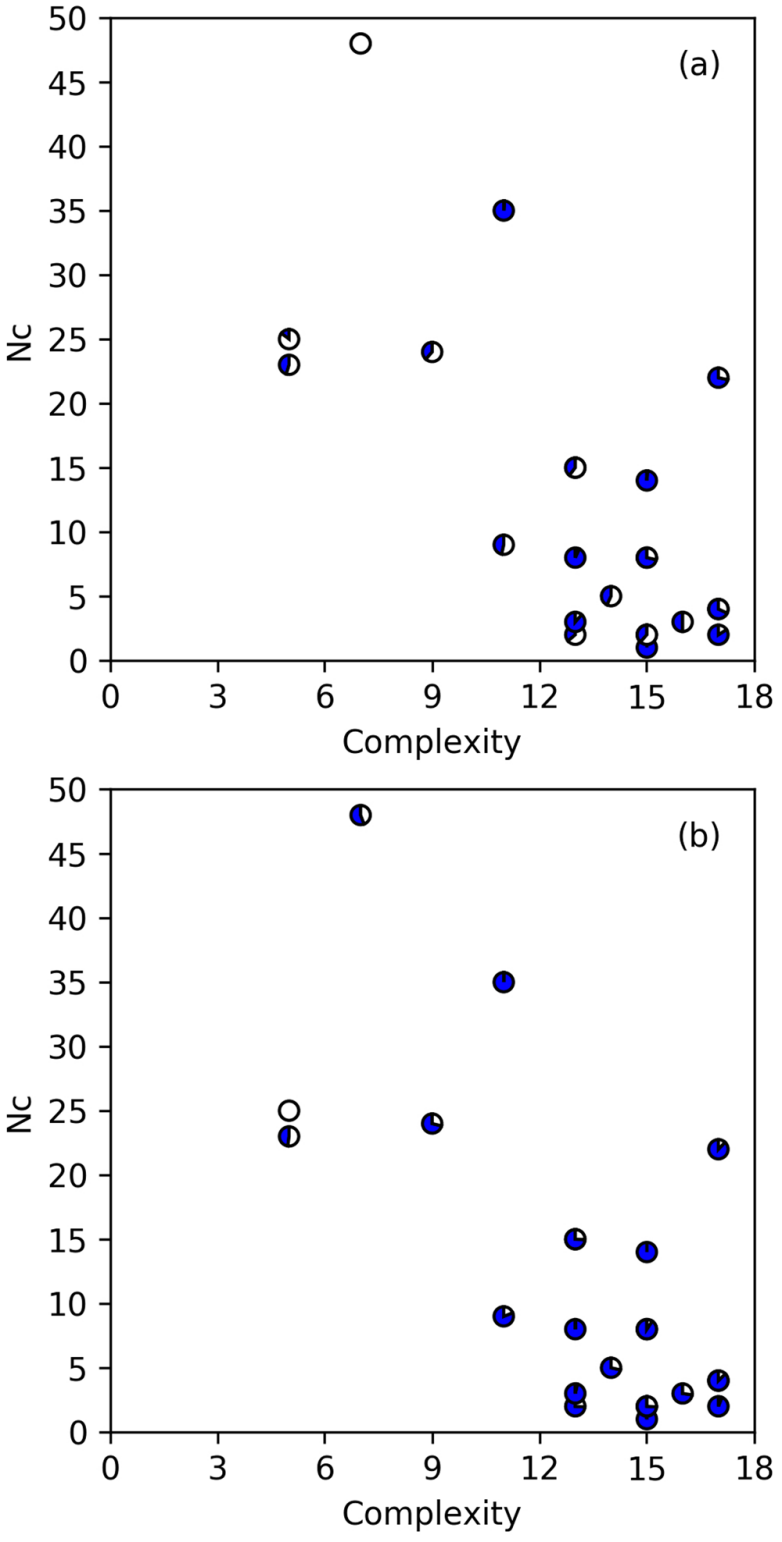
Complexity versus number of appearances (N_c_) plots after parameter re-optimization based on training and validation data. Higher coverage indicates a better model as evaluated against the test data. Zero coverage represents (**a**) the highest RMSE, 1.27 ln(MV/m) and (**b**) the lowest PCC, 0.61. Full coverage represents (**a**) the lowest RMSE, 0.39 ln(MV/m) and (**b**) the highest PCC, 0.90.

A plot of the models with complexity less than 18 on the Pareto frontiers is shown in Fig. 5, and detailed values for each entry in this plot are listed in Supplementary Table S3. We visualize each model’s performance on the test data in Fig. 5. One model, $\mathrm{ln}({F}_{b})=4.33+0.0174{E}_{g}{\omega }_{\max }$, appears on all 48 Pareto frontiers, but has relatively poor predictive performance on the test set. However, the second-most common model on the Pareto frontiers, $\mathrm{ln}({F}_{b})=\,\mathrm{ln}(5.45{E}_{g}{\omega }_{\max }+2.88)$, is one of the best-performing models. It has a lower root-mean-square error on the 82 training and validation materials than the model discovered by LASSO, and it has roughly half the root-mean-square error on the test set.

The relatively weak performance of the simpler model, $\mathrm{ln}({F}_{b})=4.33+0.0174{E}_{g}{\omega }_{\max }$ (with complexity 7), suggests that it shows up frequently because there are relatively few models to select from at that level of complexity. On the other hand, the better model, $\mathrm{ln}({F}_{b})=\,\mathrm{ln}(5.45{E}_{g}{\omega }_{\max }+2.88)$, shows up nearly as frequently and at a complexity level at which the hypothesis space of possible models is significantly larger. This suggests that one way to search for the models with the best predictive power would be to find the models that show up unusually frequently given their complexity. However this method does not resolve the issue of how to select a single model that is likely to have good predictive ability. In addition, if we repeat this exact same exercise using *F*_*b*_, rather than $\mathrm{ln}({F}_{b})$ as the output variable (see Supplementary Fig. S4 and Table S4), we find that the model that performs best on the test set shows up on Pareto frontiers less frequently than some more complex models. It would have been difficult to identify this model as being particularly promising using this first strategy.

**Figure 6:**
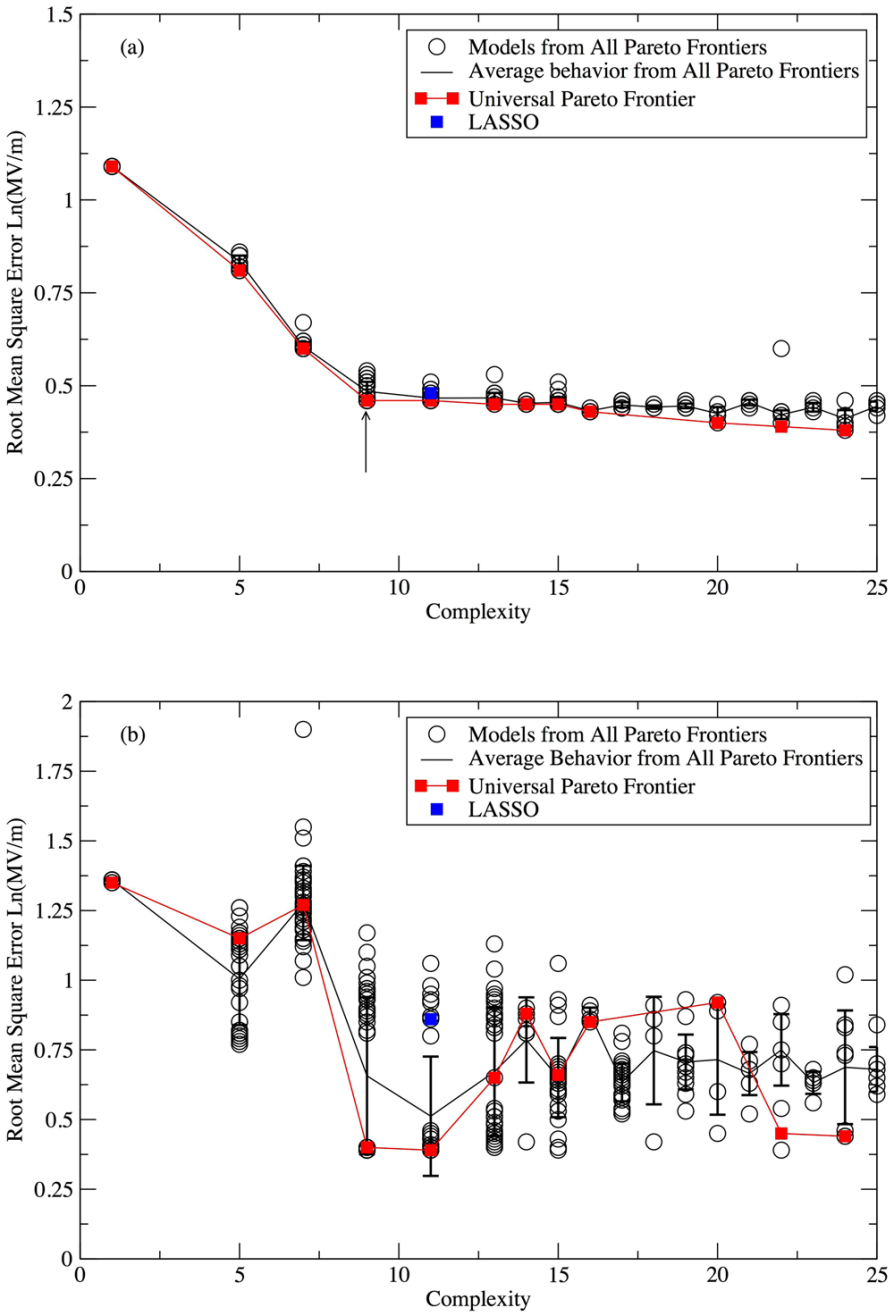
Comparison of RMSE performance of models on (**a**) training + validation and (**b**) test data between LASSO, the universal Pareto frontier and models from all Pareto frontiers. The arrow in (**a**) indicates the point at which there is a relatively large change in the slope along the Pareto frontier.

The second strategy we used is to create a “universal” Pareto frontier by combining the best models from 48 Pareto frontiers of all Eureqa runs, as evaluated against the validation data (Fig. 6a). The data used to generate this plot is provided in Table S5. When the models in the universal Pareto frontier are benchmarked against test data, we find that at some complexity values (e.g., 9 and 11), the models on the universal Pareto frontier simultaneously have the lowest (or near-lowest) RMSE for both training and test data. However at most complexity values, the best model on the training data is not the best model for the test data. The RMSE on the test data for models on the universal Pareto frontier is similar to the average RMSE over all sixteen Pareto frontiers generated using RMSE as the fitness metric, suggesting that simply appearing on the universal Pareto frontier is not an indicator of low prediction error.

**Figure 7:**
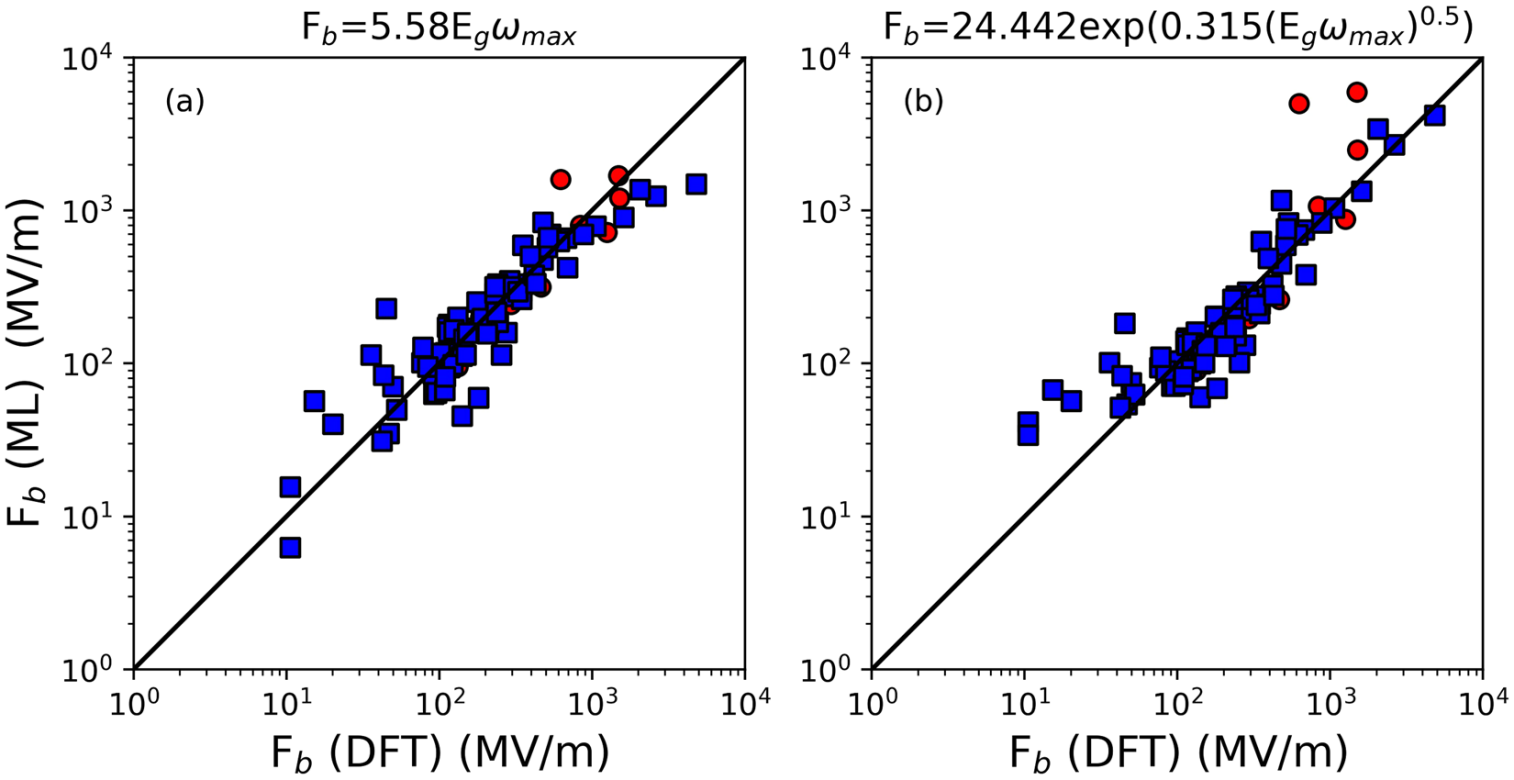
Dielectric breakdown strength F_b_ predicted by machine learning and density functional theory (DFT) for (**a**) model with complexity 9 at Universal Pareto Frontier, (**b**) the LASSO solution. Blue squares represent the prediction for training and validation data and red circles represent the prediction for test data. The black solid line indicates a perfect match between machine learning and DFT.

There does appear to be an advantage to using the universal Pareto frontier. There is a large change in slope at a complexity value of 9, which is roughly the optimal complexity value. Beyond this point, the models get only slightly better even as they get significantly more complex. The entries on the universal Pareto frontier at a complexity of 9 and 11 are also two of the best-performing models on the test data (Fig. 6b). A parity plot of the performance of the model with complexity 9 (i.e., $\mathrm{ln}({F}_{b})=1.72+\,\mathrm{ln}({E}_{g})+\,\mathrm{ln}({\omega }_{\max })$ or ${F}_{b}=5.58{E}_{g}{\omega }_{\max }$) against the LASSO solution for test, training, and validation data is provided in Fig. 7. A similar result was found when *F*_*b*_, rather than $\mathrm{ln}({F}_{b})$, was used as the objective function (see Supplementary Fig. S5 and Table S6). There is a large change in slope on the universal Pareto frontier at a complexity of 10, and the model at this point on the frontier performs very well on the test data (see Supplementary Fig. S5).

It may be fortuitous that the models at the point where the slope changes on the Pareto frontier also happen to perform very well on the test data, as models at other complexities do not perform as well (Fig. 6b). In addition, it may not always be clear what constitutes a “large change in slope.” However this change in slope may be an indication of an optimal (or near-optimal) level of complexity. The relatively rapid decrease in the error with increasing complexity up to this point may be an indication that the error is decreasing primarily because of improving model skill. Similarly, the relatively slow decrease in error at high levels of complexity may be an indication that the error is decreasingly primarily by chance; i.e. the increasing size of the hypothesis space makes it easier to find a model that happens to do well on the training and validation data but generalizes poorly.

### Predicting dielectric breakdown strength

Using the analysis in the previous section, we can now identify models that are likely to be useful as predictive models of dielectric breakdown strength. Here we report results on predicting the dielectric breakdown strength itself, rather than the natural logarithm of the breakdown strength. This choice effectively places greater importance on making accurate predictions for materials that have a high dielectric breakdown strength, and these are the materials that are often of the most technological interest. We have included results for the natural logarithm of the dielectric breakdown strength in the supporting information (Supplementary Fig. S8 and Supplementary Table S7).

**Figure 8:**
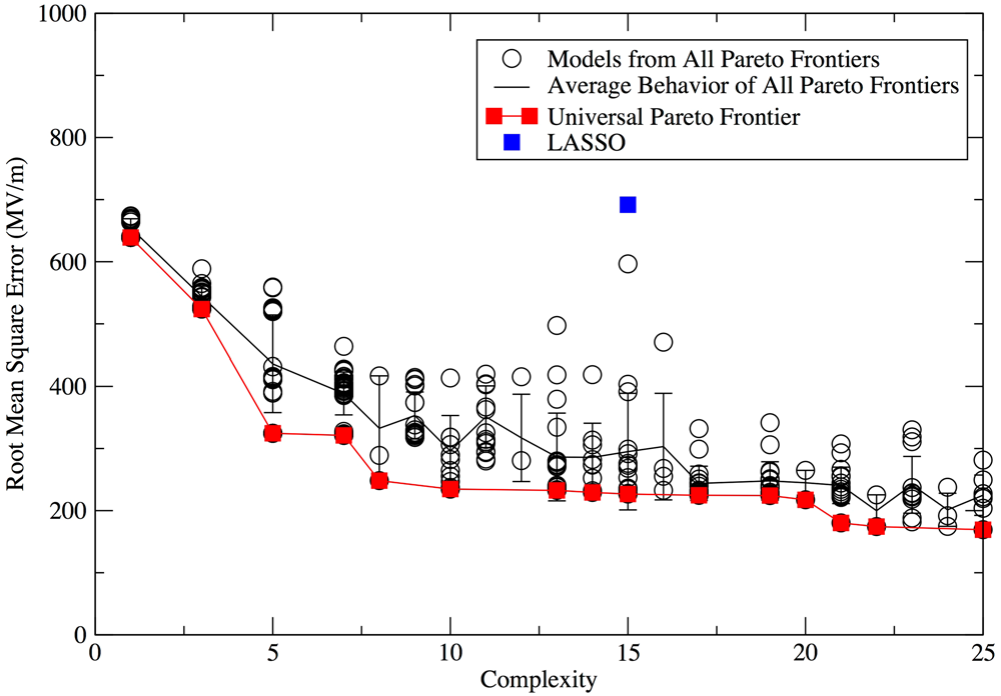
RMSE performance of models on all data (i.e., training + validation + test) for LASSO, the universal Pareto frontier, and models from all Pareto frontiers trained on all data when using dielectric breakdown strength as the output value.

To identify the models that are likely to make accurate predictions, we have used the universal Pareto frontier approach, this time including all data (i.e., training + validation + test data). For comparison, we have also included the LASSO solution, although we note that the LASSO solution was selected without considering the test set and was fit to the natural logarithm of the dielectric breakdown strength, so a direct comparison is not as straightforward as the comparisons in previous section. All models on this Pareto frontier, as well as their performance, are summarized in Table 2. The universal Pareto frontier (Fig. 8) gives a large slope change around complexity 8. Based on the analysis in the previous section, this suggests that models at about this level of complexity may have the greatest predictive power.

**Table 2:** The performance in predicting dielectric breakdown strength on all data *ε*_*al*_, training data *ε*_*tr*_, and validation data *ε*_*va*_ for models on the universal Pareto frontier constructed using training, validation, and test data.

Complexity	Model	Benchmark	*ε*_*al*_	*ε*_*tr*_	*ε*_*va*_
1	395	RMSE	639	627 ± 148	614 ± 157
		PCC	N/A	N/A	N/A
3	34.7 *ω*_max_	RMSE	524	492 ± 183	487 ± 188
		PCC	0.58	0.63 ± 0.17	0.63 ± 0.17
5 (S1)	${E}_{g}^{2}{\omega }_{\max }$	RMSE	325	321 ± 48	320 ± 48
		PCC	0.87	0.86 ± 0.05	0.85 ± 0.07
7	${E}_{g}^{2}({\omega }_{\max }-1.17)$	RMSE	321	319 ± 51	315 ± 53
		PCC	0.87	0.86 ± 0.05	0.86 ± 0.06
8 (S2)	$\frac{262{\omega }_{\max }}{14.6-{E}_{g}}$	RMSE	248	248 ± 51	238 ± 51
		PCC	0.92	0.91 ± 0.05	0.91 ± 0.05
10 (S3)	$\frac{348{\omega }_{\max }}{15-{E}_{g}}-101$	RMSE	235	235 ± 45	226 ± 43
		PCC	0.93	0.91 ± 0.05	0.92 ± 0.05
13	$0.00399{e}^{{E}_{g}}+5.84{E}_{g}{\omega }_{\max }$	RMSE	233	231 ± 50	223 ± 49
		PCC	0.93	0.92 ± 0.05	0.92 ± 0.05
14	$\frac{184}{2.11{\omega }_{\max }-41.3}+5.92{E}_{g}{\omega }_{\max }$	RMSE	229	227 ± 50	221 ± 48
		PCC	0.93	0.92 ± 0.05	0.92 ± 0.05
15	$4.81\ast {10}^{-9}{e}^{{E}_{g}}+6.11{E}_{g}{\omega }_{\max }$	RMSE	227	227 ± 49	217 ± 47
		PCC	0.94	0.92 ± 0.05	0.92 ± 0.05
17	$0.00649{e}^{{E}_{g}}+20.5{\omega }_{\max }\,\mathrm{ln}({E}_{g})$	RMSE	225	226 ± 44	215 ± 44
		PCC	0.94	0.92 ± 0.04	0.93 ± 0.05
19	$0.0046{e}^{{E}_{g}}+16.6{\omega }_{\max }\sqrt{{E}_{g}}-80$	RMSE	224	226 ± 43	214 ± 42
		PCC	0.94	0.92 ± 0.04	0.93 ± 0.05
20	$$\begin{array}{c}0.00416{e}^{{E}_{g}}+5.67{E}_{g}{\omega }_{\max }\\ +21.8/(5.54-{E}_{g})\end{array}$$	RMSE	217	219 ± 37	210 ± 35
		PCC	0.94	0.93 ± 0.04	0.93 ± 0.05
21	$$\begin{array}{c}5.92{E}_{g}{\omega }_{\max }+138/(40.6-{\omega }_{\max })\\ +184/(2.11{\omega }_{\max }-41.3)\end{array}$$	RMSE	189	185 ± 38	165 ± 42
		PCC	0.96	0.95 ± 0.04	0.96 ± 0.03
22	$$\begin{array}{c}0.0003{E}_{g}{e}^{{E}_{g}}+5.23{E}_{g}{\omega }_{\max }\\ +214/(40.7-{\omega }_{\max })\end{array}$$	RMSE	174	184 ± 32	156 ± 39
		PCC	0.96	0.95 ± 0.04	0.96 ± 0.03
25	$$\begin{array}{c}15{\omega }_{\max }+0.0047{E}_{g}^{3}{\omega }_{\max }^{2}\\ -1.12\ast {10}^{-13}{e}^{{\omega }_{\max }-{E}_{g}}\end{array}$$	RMSE	169	165 ± 38	166 ± 36
		PCC	0.96	0.96 ± 0.03	0.95 ± 0.03
15 (S4) (LASSO)	$24.442{e}^{0.315\sqrt{{E}_{g}{\omega }_{\max }}}$	RMSE	692	674 ± 242	614 ± 259
		PCC	0.74	0.78 ± 0.08	0.79 ± 0.10

**Figure 9:**
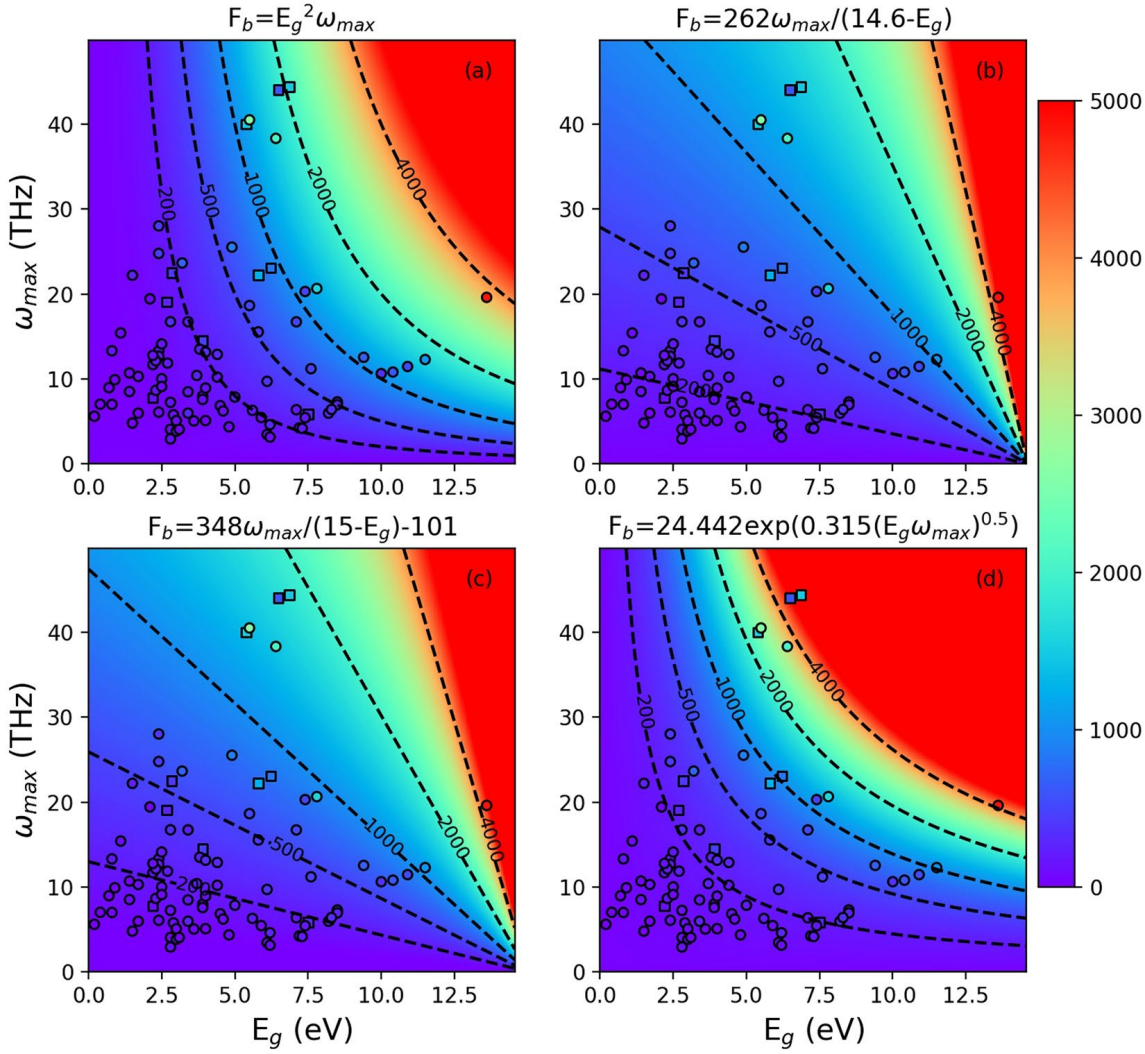
Contour plot of dielectric breakdown strength F_b_ predicted by machine learning along with scatter plot of the values calculated by density functional theory (DFT) for (**a**) solution S1, (**b**) solution S2, (**c**) solution S3 and (**d**) solution S4 (the LASSO solution). The circles are training and validation data, and the squares are test data. The spheres and squares share the same color-coding scheme as the contour plot. The black dashed lines indicate contour levels labeling machine-learning-predicted F_b_ values at 200, 500, 1000, 2000, 4000 MV/m.

We have selected from the universal Pareto frontier three models with complexity around 8, labeled S1, S2, and S3 in Table 2, for further analysis. We have also included the LASSO solution for comparison, and label it S4. Parity plots of the values predicted by the models vs. the values predicted by DFT for these four models are provided in Supplementary Figs S9–S11. We emphasize that because the models S1, S2, and S3 were optimized for *F*_*b*_ and the LASSO solution was optimized for $\mathrm{ln}({F}_{b})$, these plots should not be used to compare the performance of genetic programming vs. LASSO (that comparison was discussed in the previous section). To better understand how each of these four model predicts how *F*_*b*_ will change as a function of *E*_*g*_ and *ω*_max_, we have created plots showing the predicted vs. DFT-calculated values as a function of *E*_g_ and *ω*_max_ (Fig. 9). These plots make it clear that although there is a dependence on *E*_*g*_ and *ω*_max_, these variables are not sufficient for a complete description, as there are several pairs of materials in which both materials have similar values for *E*_*g*_ and *ω*_max_ but very different breakdown strengths.

Of particular interest are models S2 and S3, as these are arguably the best models on the entire set of data in terms of combined simplicity and accuracy. Models S2 and S3 include terms with $14.6-{E}_{g}$ and $15-{E}_{g}$ in their denominators, respectively. These terms will have singularities for materials with band gaps of 14.6 or 15 eV and will become negative for materials with larger band gaps. Fortunately, apart from condensed noble gasses, there is no known material with a band gap larger than that of LiF^56,57^, which has a calculated band gap of 13.6 eV. Thus the upper bound on the band gaps in these models corresponds well to the known upper limit in nature for band gaps of materials of the type considered here. Models with similar form showed up on the universal Pareto frontier generated using just training and validation data (Supplementary Table S6), and their persistence when test data was included suggests they have true predictive power. If we generate a universal Pareto frontier using only models discovered using the training and validation data, but with error (the y-axis) evaluated against all data, the models with forms similar to S2 and S3 have the best performance (Supplementary Fig. S12 and Supplementary Table S8).

Models S2 and S3, and similar models, are also of interest because they exist outside of the hypothesis space that was searched by the LASSO algorithm^48^. This highlights a problem with methods that attempt to enumerate all possible solutions in the hypothesis space: the combinatorial space of even relatively simple functions is very large and difficult to comprehensively enumerate. The advantage of an approach such as genetic programming is that it can effectively search this hypothesis space without the need to explore the entire space; it naturally focuses on the regions of the space with the most promising (i.e. “fit”) solutions. As discussed in this paper, care must be taken to avoid overfitting the training data, but that will be a problem with any algorithm that searches a similarly-sized hypothesis space, including LASSO.

## Conclusion

In this paper, we demonstrated that genetic programming is an effective way to search a large hypothesis space of simple functions of known material properties. For the specific property of the dielectric breakdown strength of materials, we identified a new family of models based on ${\omega }_{\max }{( \sim 15{\rm{eV}}-{E}_{g})}^{-1}$ that performed well on the training and validation data and then again on the test data. Our results indicate that there is a substantial risk to overfitting the training and validation data, both with genetic programming and with the LASSO approach. We explored different techniques to mitigate this risk and facilitate the use of genetic programming to discover models with good predictive power. The more effective of these appears to be finding the model(s) at or near the point at which the Pareto frontier starts to level off. It can be helpful to consider the number of times a model shows up in repeated genetic programming runs, but this approach appears to be less reliable in identifying models with good predictive power. We believe further exploration of these and related approaches will make genetic programming a more practically useful tool for researchers. There are a number of additional potential areas for improvement, including how to best define “complexity” and how to best partition the known data. Despite the room for further improvement, the relative success of the genetic programming approach in identifying simple models of dielectric breakdown strength provides additional evidence that it is a valuable tool for descriptor identification in materials science and engineering.

## Electronic supplementary material

Supplementary Information
