# Supplementary material for: Identifying models of dielectric breakdown strength from high-throughput data via genetic programming
Source: Sci Rep. 2017 Dec 14;7:17594. doi: 10.1038/s41598-017-17535-3 (PMC5730619; doi:10.1038/s41598-017-17535-3)
Supplement: Supplementary file 1 — Supplementary Information [file 41598_2017_17535_MOESM1_ESM.pdf]

Supplementary Information

Identifying models of dielectric breakdown strength from high-throughput data via genetic programming

Fenglin Yuan and Tim Mueller*

Department of Materials Science and Engineering, Johns Hopkins University Baltimore MD 21218 USA

* To whom correspondence should be addressed: T. M. Email: tmueller@jhu.edu

1. Setup of Parameters for Genetic Programming

Genetic programming (GP) requires setup of key parameters before running the optimization, including target function of optimization, complexity values of operators, and the relative ratio of training versus validation data.

2. Three Measurements of Errors

Typically, we can have three different choice of target function of optimization: mean absolute error (MAE), root mean square error (RMSE) and Pearson correlation coefficient (PCC). Supposing there are two series of data of length **N**: one is $\mathbf{D_m}$, data predicated by the in genetic programming; the other is $\mathbf{D_r}$, data from reference. In order to assess the deviation of predicted data $\mathbf{D_m}$ from reference data $\mathbf{D_r}$ and therefore measure the errors, three typical functions MAE, RMSE and PCC can be constructed via Supplementary Equations S1-S3. The MAE and RMSE measure the deviation of error from reference, while PCC estimates the linear correlation between predicted and reference data.

$$\sigma_{MAE} = \frac{1}{N}\sum_{i=1}^{N}\left|D_m^i - D_r^i\right| \tag{S1}$$

$$\sigma_{RMSE} = \sqrt{\frac{1}{N}\sum_{i=1}^{N}(D_m^i - D_r^i)^2} \tag{S2}$$

$$\sigma_{PCC} = \frac{\sum_{i=1}^{N}(D_m^i - \overline{D_m})\sum_{i=1}^{N}(D_r^i - \overline{D_r})}{\sqrt{\sum_{i=1}^{N}(D_m^i - \overline{D_m})^2\sum_{i=1}^{N}(D_r^i - \overline{D_r})^2}} \tag{S3}$$

3. Correction for Non-Zero Intercept in PCC Optimization

Two data sets can be highly correlated even if their values are very different. Thus when using PCC as the metric for optimization, it is necessary to do a linear transformation of the predicted data to make it directly comparable to the data calculated using density functional theory (DFT). In Supplementary Fig. S1, we demonstrate the

effect of the linear transformation by showing one representative model $E_g\omega_{max}^2/(16-E_g)$ from our PCC runs. For PCC runs, such linear transformations are always applied before model evaluation.

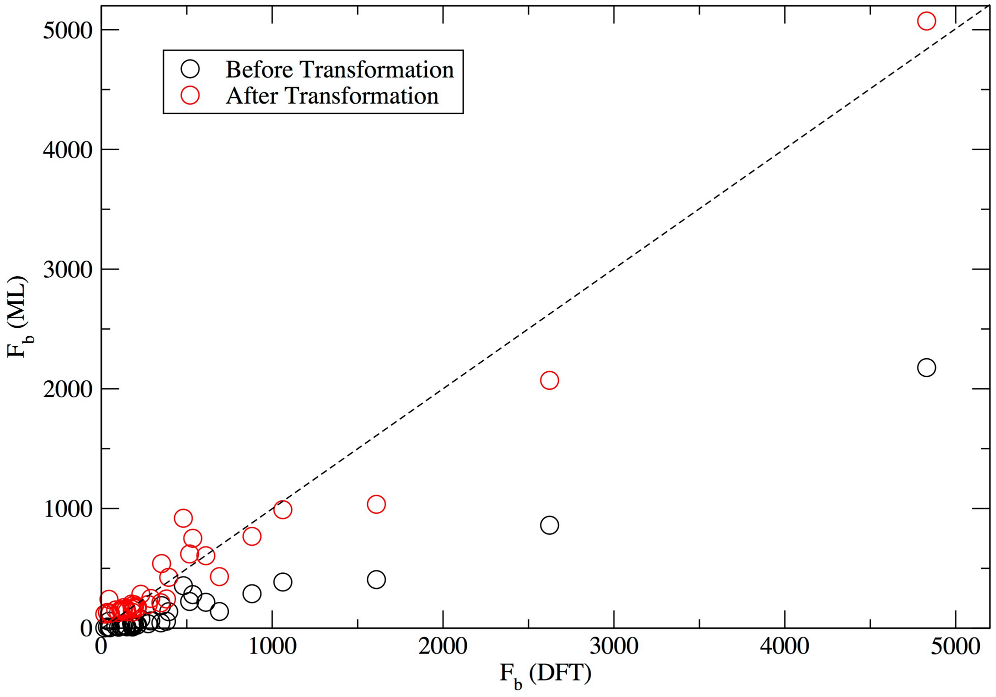

Supplementary Figure S1. Linear transformation of the model $E_g\omega_{max}^2/(16-E_g)$, discovered using PCC optimization, to make the predicted data directly comparable to the DFT data.

4. Choose Relative Ratio between training and validation data

We partitioned the training and validation structures into 50% training and 50% validation data, the default setting in Eureqa.[1] For the three objective functions, we evaluated three different ratios between training and total data (i.e., R(train)): 0.4, 0.5 and 0.6 (Supplementary Fig. S2). It's noteworthy that for all three target functions, on average the 0.5 ratio produced the most on the Pareto frontier after 4×10^{10} evaluated equations.

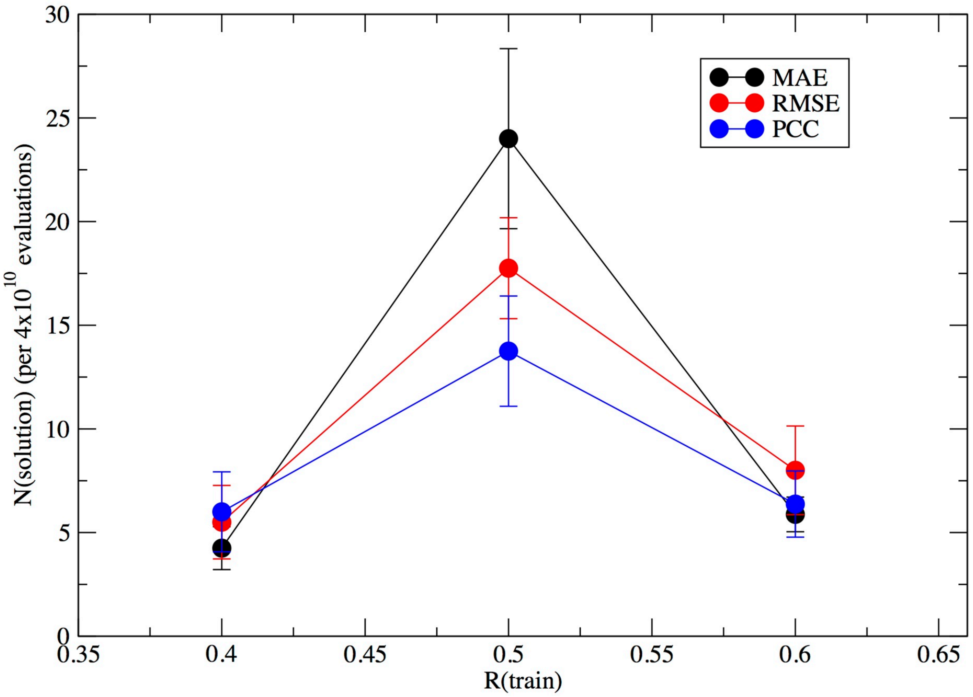

Supplementary Figure S2. The number of on the Pareto frontier after $4*10^{10}$ evaluations versus R(train), the ratio of training data to total input data. Error bars are the standard deviations from eight parallel runs.

5. Complexity Values of Operators

In this paper, we adopted the Eureqa's default values of complexity for all operators used in feature space sampling. The specific complexity of each operator is tabulated in Supplementary Table S1.

**Operators**	**Complexity**	**Operators**	**Complexity**
Plus	1	Square Root	4
Minus	1	Exponential	4
Division	2	Logarithm	4
Multiplication	1	Constant/Parameter	1

Supplementary Table S1. Table of complexity values of operators.

6. Structure Data for Crystalline Dielectric Materials (Test Set)

We used 10 crystalline dielectric materials as test set, where their crystal structures belong to 4 space groups Pm-3m, Fm-3m, Cm and P1 (Supplementary Table S2). The crystal structures in the test set are different from training and validation sets, which provide the necessary condition to perform out-of-range test and ensure the general assessment of models. The data for the ten structures in the test set is from two published papers by Kim et al.[2,3]

**Name**	**F_b (MV/m)**	**E_g (eV)**	**ω_{max} (THz)**	**Space Group**
$BaSnO_3$	259.7003	2.67	19.06	Pm-3m
$CaGeO_3$	401.3308	2.84	22.5	Pm-3m
$CaSiO_3$	833.8868	6.22	23.07	Pm-3m
$BSiO_2F$	1507.0481	5.39	40.03	P1
$BaBO_2F$	623.6073	6.49	44.07	Cm
$SrBO_2F$	1491.3854	6.86	44.40	Cm
Li_2S	462.5	3.9	14.488	Fm-3m
Na_2S	132.7	2.2	7.765	Fm-3m
$SrCl_2$	293.4	7.5	5.822	Fm-3m
ZrO_2	1253.0	5.8	22.231	Fm-3m

Supplementary Table S2. Information of 10 crystalline materials in test set.

7. Correlation Between Features and Target

To identify linear relationships between the 8 feature properties and dielectric breakdown strength, we have calculated the Pearson correlation coefficients using the 82 structures in the training and validation sets. Our results were visualized via color-coded pie plot in Supplementary Fig. S3, where the covered area indicates the absolute value of PCC, colors the sign of PCC (blue is positive and red is negative).

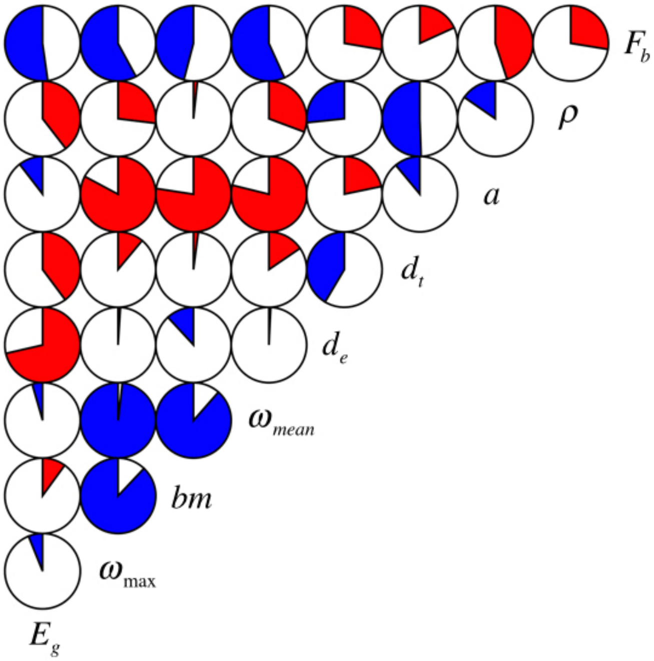

Supplementary Figure S3. Pearson correlation map for eight features and dielectric breakdown strength. The colors indicate the numeric sign of PCC values (red as negative; blue as positive); the covered areas indicate the absolute magnitudes of PCC values.

8. Counting the number of times models appear

Often Eureqa will return two different models that are effectively the same, with only the constant parameters slightly differing. To count the number of times each model appears on the Pareto frontiers generated by Eureqa, we re-optimized the constant parameters to find the set of at the local minimum for the RMSE as evaluated against all training data. We used a BFGS local minimization algorithm in liblbfgs library wrapped within pylbfgs package.[4,5] The re-optimized parameters are used in Supplementary Table S3, Table S4, Fig. S4, and Fig. 5. We note that this parameters re-optimization slightly improved the quality of the results. For example, in Supplementary Table S4, Eureqa generates a set of models similar to $277\omega_{\max}/(14.7-E_g)$, with Pearson correlation coefficients (against all training and test data) of 0.91-0.93. After parameter re-optimization, the model $316.1\omega_{\max}/(14.9-E_g)$ is found with a Pearson correlation coefficient of 0.95.

In Supplementary Table S3, we show various metrics for different models that were found when the logarithm of the dielectric breakdown strength was used as the output value. These data are plotted in Fig. 5 in the main text. In Supplementary Table S4 and Fig. S4, similar data are shown for models discovered using the dielectric breakdown strength as the output value.

Several of the models found using the logarithm of the dielectric breakdown strength include a $\ln(E_g\omega_{\max})$ term, indicating that $E_g\omega_{\max}$ is a simple, useful descriptor of the dielectric breakdown strength. These models include $\ln(5.4E_g\omega_{\max}+0.54\omega_{\max})$, $\ln(5.45E_g\omega_{\max}+2.88)$, $1.7+1.01(\ln E_g+\ln\omega_{\max})$, $1.14\ln(12+5.48E_g\omega_{\max})-0.8$, $\ln E_g+\ln\omega_{\max}+\ln(4.9+0.065\omega_{\max})$, and $1.77+0.97\ln E_g+\ln\omega_{\max}$.

N_c	Complexity	Model	σ_{at}	σ_{tt}	p_{at}	p_{tt}
23	5	$4.16+0.095\omega_{\max}$	0.85	0.87	0.63	0.75
25	5	$4.08+0.256E_g$	0.81	1.14	0.66	0.61
48	7	$4.33+0.0174E_g\omega_{\max}$	0.60	1.27	0.84	0.77
24	9	$3.15+0.24E_g+0.09\omega_{\max}$	0.50	0.93	0.89	0.81
9	11	$3.2+0.314(E_g\omega_{\max})^{0.5}$	0.48	0.86	0.91	0.84
35	11	$\ln(5.45E_g\omega_{\max}+2.88)$	0.46	0.40	0.91	0.90
15	13	$\ln(5.4E_g\omega_{\max}+0.54\omega_{\max})$	0.46	0.40	0.91	0.90
15	13	$3.1+0.0875\omega_{\max}+0.93\ln(E_g)$	0.46	0.92	0.91	0.82
2	13	$2.12+1.05(0.0842\omega_{\max}+E_g^{1/2})$	0.46	0.95	0.91	0.82
3	13	$\ln E_g+\ln\omega_{\max}+\ln(4.9+0.065\omega_{\max})$	0.46	0.49	0.91	0.89
8	13	$1.14+1.14\ln(E_g\omega_{\max}+2.19)$	0.46	0.45	0.91	0.90
5	14	$3.56+0.196E_g+0.087\omega_{\max}-\frac{0.422}{E_g}$	0.45	0.89	0.91	0.82
14	15	$1.7+1.01(\ln E_g+\ln\omega_{\max})$	0.46	0.40	0.91	0.90
1	15	$1.77+0.97\ln E_g+\ln\omega_{\max}$	0.46	0.39	0.91	0.90
8	15	$1.06+1.69(E_g\omega_{\max})^{1/4}$	0.45	0.62	0.91	0.88

2	15	$1.14\ln(12+5.48E_g\omega_{\max})-0.8$	0.46	0.45	0.91	0.90
8	15	$-0.65+0.92(\ln E_g$ $+\ln(60+\omega_{\max}^2))$	0.44	0.64	0.91	0.87
2	15	$0.64(0.137(E_g+\omega_{\max})$ $+\ln E_g)+3.05$	0.45	0.93	0.91	0.82
3	16	$5.3+0.0113E_g\omega_{\max}-$ $20/(5.3+E_g\omega_{\max})$	0.43	0.83	0.92	0.82
22	17	$1.91+0.94\ln E_g+0.66\omega_{\max}^{1/2}$	0.44	0.64	0.91	0.86
4	17	$2.28+0.0056E_g\omega_{\max}+$ $0.76(\ln E_g+\ln\omega_{\max})$	0.43	0.70	0.92	0.85
4	17	$0.94+1.07E_g^{1/2}+0.67\omega_{\max}^{1/2}$	0.45	0.67	0.91	0.86
2	17	$-0.095+0.822(\ln E_g+$ $\ln(37.55+E_g\omega_{\max}+\omega_{\max}^2))$	0.44	0.53	0.91	0.88

Supplementary Table S3. The number of appearances N_c, Complexity, Model, RMSE Error σ and PCC p for all training (at) and testing data (tt) after parameter re-optimization based on all training data using the natural logarithm of dielectric breakdown strength as the output value.

N_c	Complexity	Model	σ_{at}	σ_{tt}	p_{at}	p_{tt}
38	3	$37.7\omega_{\max}$	538	416	0.579	0.726
6	3	$\omega_{\max}^2$	558	529	0.596	0.685
18	5	$8.84E_g\omega_{\max}$	350	742	0.863	0.752
11	5	$E_g^2\omega_{\max}$	298	489	0.889	0.717
2	5	$118.6E_g - 173.4$	548	520	0.522	0.532
1	7	$16\omega_{\max} + \omega_{\max}^2$	516	817	0.600	0.697
1	7	$E_g\omega_{\max} + \omega_{\max}^2$	511	620	0.667	0.695
14	7	$11.63E_g\omega_{\max} - 45.61E_g$	326	990	0.864	0.745
18	8	$316.1\omega_{\max} / (14.9 - E_g)$	222	387	0.947	0.741
10	9	$6.02E_g\omega_{\max} + 0.13E_g\omega_{\max}^2$	333	1132	0.862	0.716
22	11	$87.1 + 0.05(E_g\omega_{\max})^2$	215	1562	0.943	0.650
11	11	$121\exp(0.0134E_g\omega_{\max})$	186	2360	0.957	0.558
11	12	$2.91E_g\omega_{\max}^2 / (16.75 - E_g)$	154	1255	0.980	0.661
11	13	$160\exp(0.0123E_g\omega_{\max}) - 65.5$	185	2245	0.958	0.570
12	14	$2.55E_g\omega_{\max}^2 / (16.4 - E_g) + 99.7$	127	1135	0.980	0.660
28	15	$0.275\omega_{\max}^2\exp(0.279E_g) + 85.1$	131	1146	0.979	0.655

Supplementary Table S4. The number of appearances N_c, Complexity, Model, RMSE Error σ and PCC p for all training (at) and testing data (tt) after parameter re-optimization based on all training data using the dielectric breakdown strength as the output value.

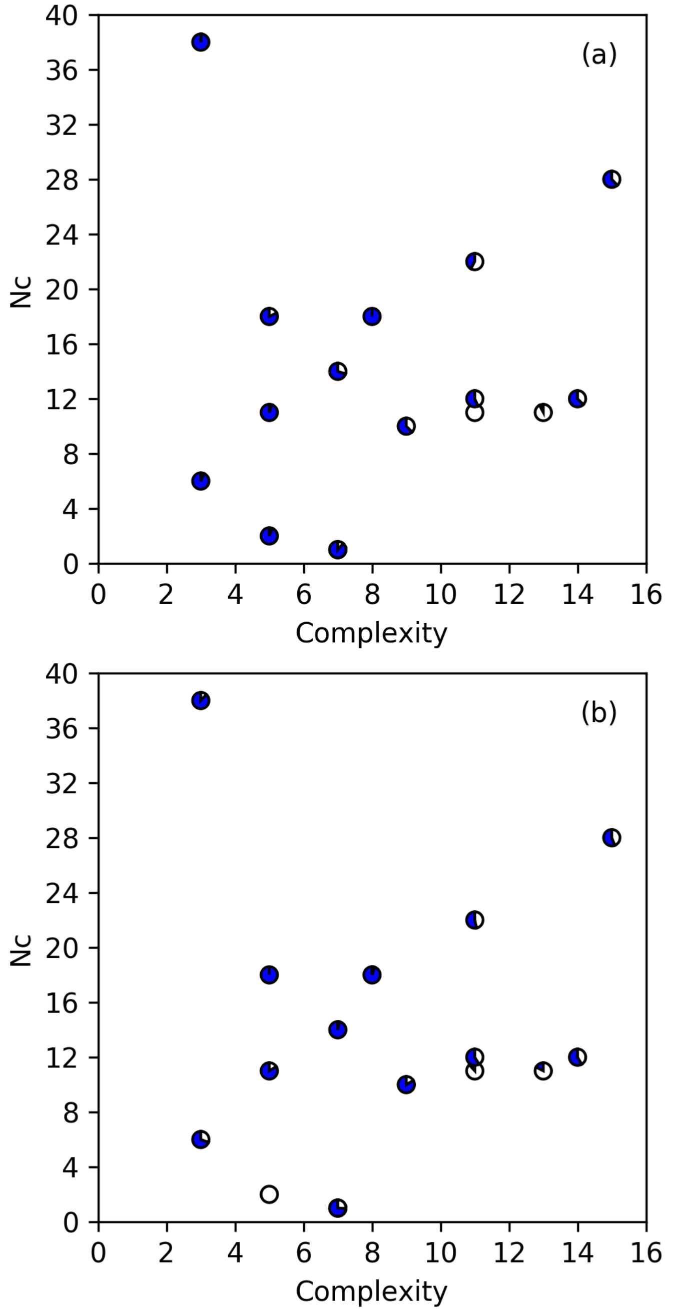

Supplementary Figure S4. Complexity versus number of appearances (Nc) plots after parameter re-optimization based on all training and validation data, using the dielectric breakdown strength as the output value. A higher coverage indicates a better model as evaluated against the test data. Zero coverage represents the highest RMSE equal to 2402 MV/m (a) and the lowest PCC equal to 0.53 (b). Full coverage represents the lowest RMSE equal to 386 MV/m (a) and the highest PCC equal to 0.75 (b).

9. Universal Pareto Frontiers using training and validation data

Supplementary Tables S5 and S6 show all found for the universal Pareto frontiers generated using only the training and validation data. The corresponding figures are Fig. 6 in the main text and Supplementary Fig. S5.

Complexity	Model	σ_{at}	σ_{tt}	p_{at}	p_{tt}
1	5.21	1.09	1.35	N/A	N/A
5	$4.1+0.26E_g$	0.81	1.15	0.66	0.61
7	$4.33+0.017E_g\omega_{\max}$	0.60	1.27	0.84	0.77
9	$1.72+\ln E_g+\ln\omega_{\max}$	0.46	0.40	0.91	0.90
11	$\ln(2.68+5.52E_g\omega_{\max})$	0.46	0.39	0.91	0.90
13	$\ln E_g+\ln(24+0.25\omega_{\max}^2)$	0.45	0.65	0.91	0.87
14	$3.6+0.196E_g$ $+0.086\omega_{\max}-0.41/E_g$	0.45	0.88	0.91	0.82
15	$1.84+\ln E_g+0.67\omega_{\max}^{1/2}$	0.45	0.66	0.91	0.87
16	$5.3+0.012E_g\omega_{\max}$ $-19.8/(5.3+E_g\omega_{\max})$	0.43	0.85	0.92	0.82
20	$0.0873\omega_{\max}+0.856\ln E_g$ $-0.113/(19.5-\omega_{\max})+3.23$	0.40	0.92	0.93	0.83
22	$1.01+1.1\ln(\omega_{\max}+E_g\omega_{\max}$ $-18.4/(2.1\omega_{\max}-E_g\omega_{\max}))$	0.39	0.45	0.94	0.84
24	$0.8+1.1\ln(\omega_{\max}+E_g\omega_{\max}$ $+E_g-\frac{18.4}{2.1\omega_{\max}-E_g\omega_{\max}})$	0.38	0.44	0.94	0.84
11 (LASSO)	$3.196+0.315\sqrt{E_g\omega_{\max}}$	0.48	0.86	0.90	0.84

Supplementary Table S5. Complexity, Model, RMSE Error σ and PCC p for all training

(at) and testing data (tt) for models on the universal Pareto frontier generated using only the training and validation data. The output value was the natural logarithm of dielectric breakdown strength. LASSO model is included for comparison.

Complexity	Model	σ_{at}	σ_{tt}	p_{at}	p_{tt}
1	354	643.1	617.2	N/A	N/A
3	$37.7\omega_{\max}$	537.6	416.2	0.58	0.73
5	$E_g^2\omega_{\max}$	298.4	488.5	0.89	0.72
7	$E_g^2\omega_{\max} + 6.83\omega_{\max}$	281.9	580.8	0.90	0.73
8	$277\omega_{\max} / (14.7 - E_g)$	227.5	351.4	0.94	0.74
10	$14.5\omega_{\max}^2 / (14.7 - E_g)$	172.0	1177.7	0.97	0.68
11	$\omega_{\max} e^{0.397 E_g} + \omega_{\max}^2$	167.2	743.8	0.97	0.70
12	$\frac{11.7(\omega_{\max}^2 + \omega_{\max} E_g)}{(14.7 - E_g)}$	139.7	963.4	0.98	0.69
14	$101 + 2.55\frac{E_g\omega_{\max}^2}{16.4 - E_g}$	126.7	1139.3	0.98	0.66
16	$96 + E_g + 2.53\frac{E_g\omega_{\max}^2}{16.4 - E_g}$	126.4	1127.8	0.98	0.66
17	$0.004e^{E_g} + 17.7E_g + 0.25E_g\omega_{\max}^2$	121.4	1077.1	0.98	0.68
19	$0.004e^{E_g} + 10.5E_g + 0.24E_g\omega_{\max}^2 + 47$	120.6	1028.8	0.98	0.68
21	$0.236E_g\omega_{\max}^2 + \frac{1090}{13.9 - E_g} + \frac{142}{\omega_{\max} - 20.5}$	97.7	1018.9	0.99	0.69
23	$0.238E_g\omega_{\max}^2 + \frac{1080}{13.9 - E_g} + \frac{92.2}{\omega_{\max} - 20.5} - 14.2$	84.5	1023.3	0.99	0.69

28	$0.0043e^{E_g} + 13.8E_g + 37.6$ $+0.24E_g\omega_{\max}^2 - \frac{88}{20.5 - \omega_{\max}}$	82.3	1040.6	0.99	0.69

Supplementary Table S6. Complexity, Model, RMSE Error σ and PCC p for all training (at) and testing data (tt) for models on the universal Pareto frontier generated using only the training and validation data. The output value was the dielectric breakdown strength.

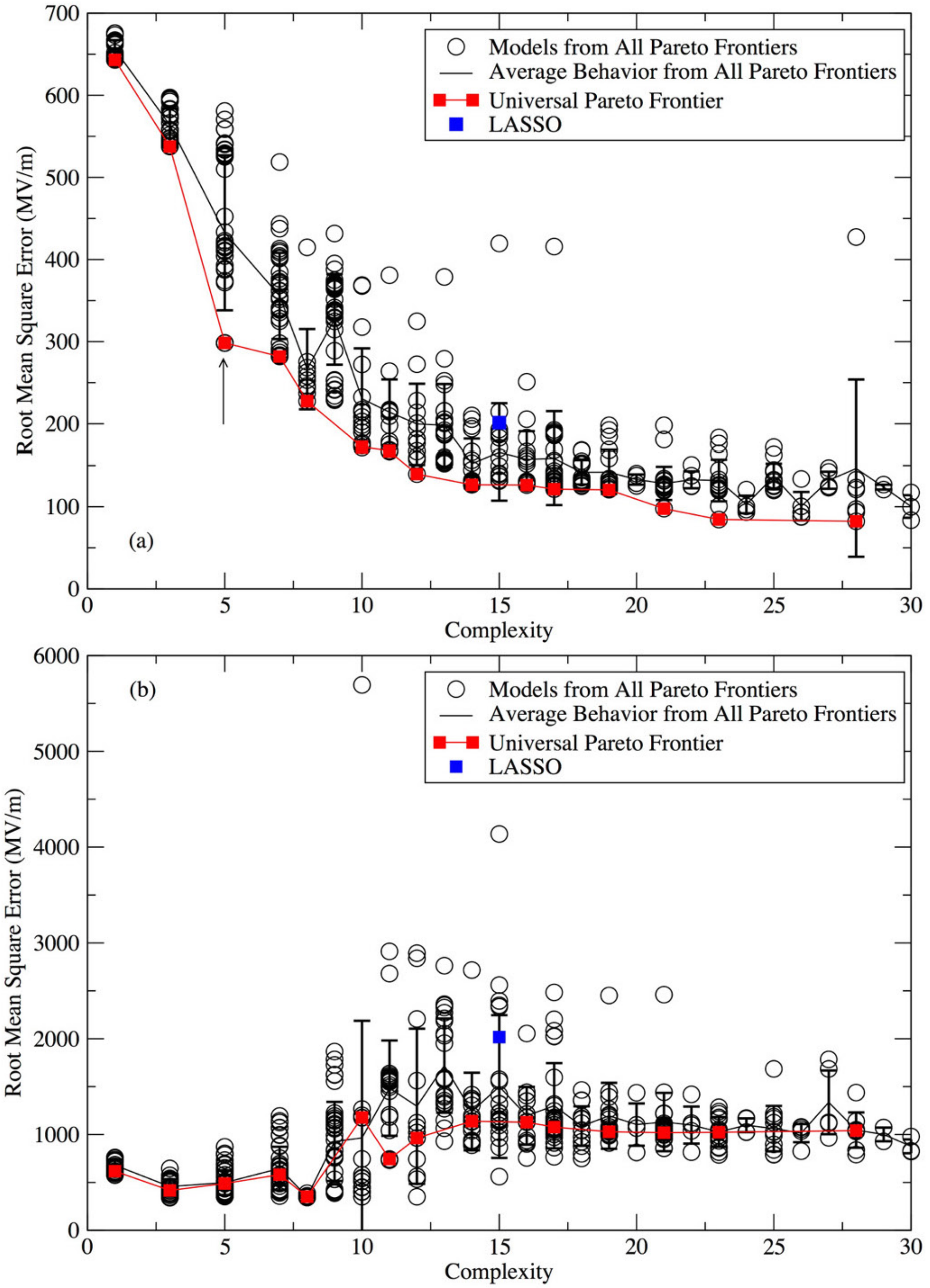

Supplementary Figure S5. RMSE performance of models on (a) all training (i.e., training+validation) and (b) test data for the LASSO model, models on the universal Pareto frontier and models from all Pareto frontiers. The output value for these runs was the dielectric breakdown strength.

10. Output of the genetic programming models when using the dielectric breakdown strength as the output value

Supplementary Figure S6 shows the performance of the 48 Eureqa model runs that used the dielectric breakdown strength as the output value. The LASSO solution is also shown for reference, but the LASSO solution was fit to the logarithm of the dielectric strength rather than the dielectric breakdown strength, so these plots should not be interpreted as a comparison of the qualities of the found by the different machine learning approaches.

In Supplementary Fig. S7, the quality of the predictions on the validation and test data is shown. Supplementary Figs. S6 and S7 are comparable to Figs. 3 and 4 in the main text.

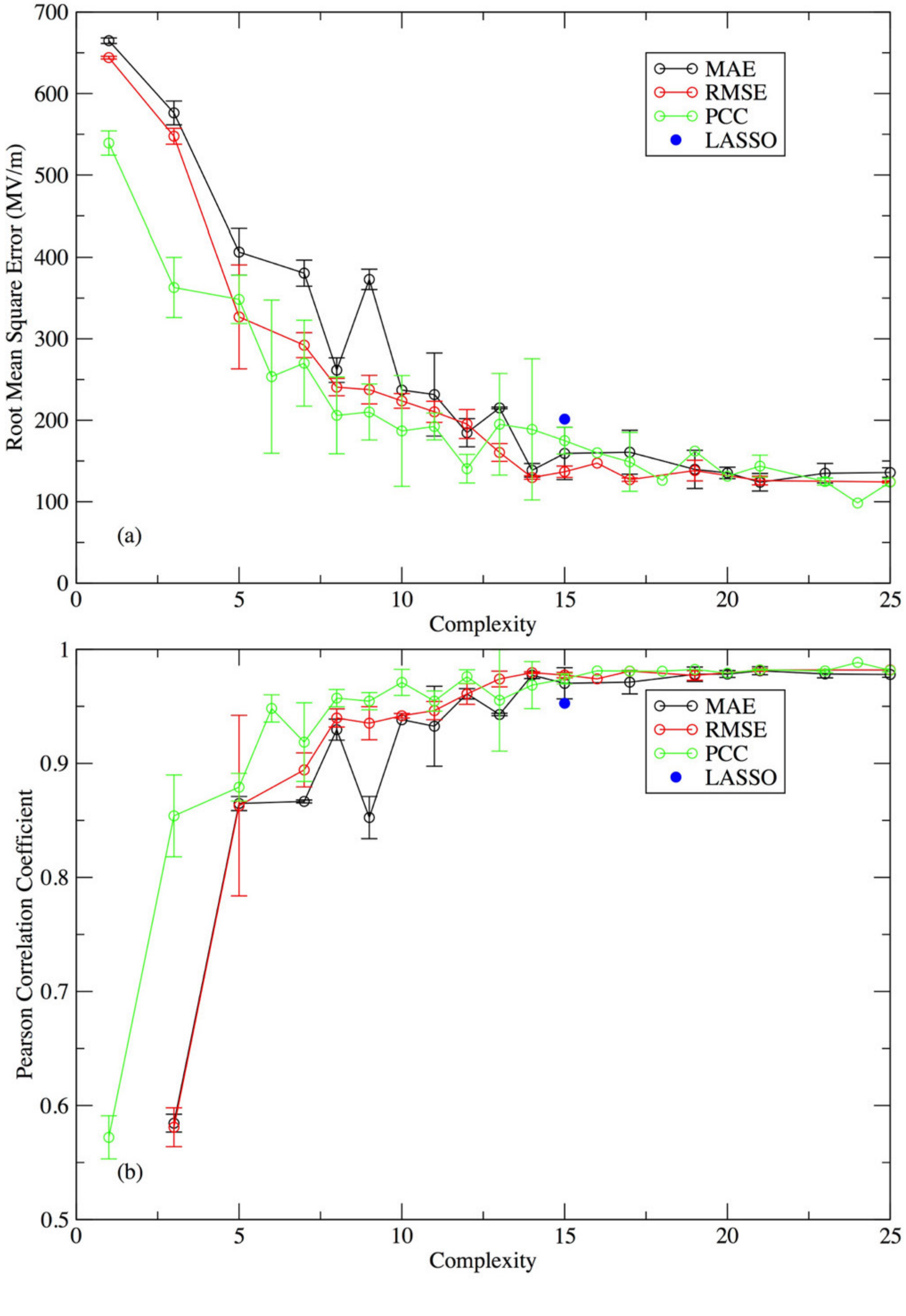

Supplementary Figure S6. (a) RMSE and (b) PCC performance on total input data (training plus validation datasets) after MAE, RMSE, PCC optimizations. The LASSO solution is also shown. The error bar is calculated using the standard deviation from 16 parallel runs. The output value for these runs was the dielectric breakdown strength.

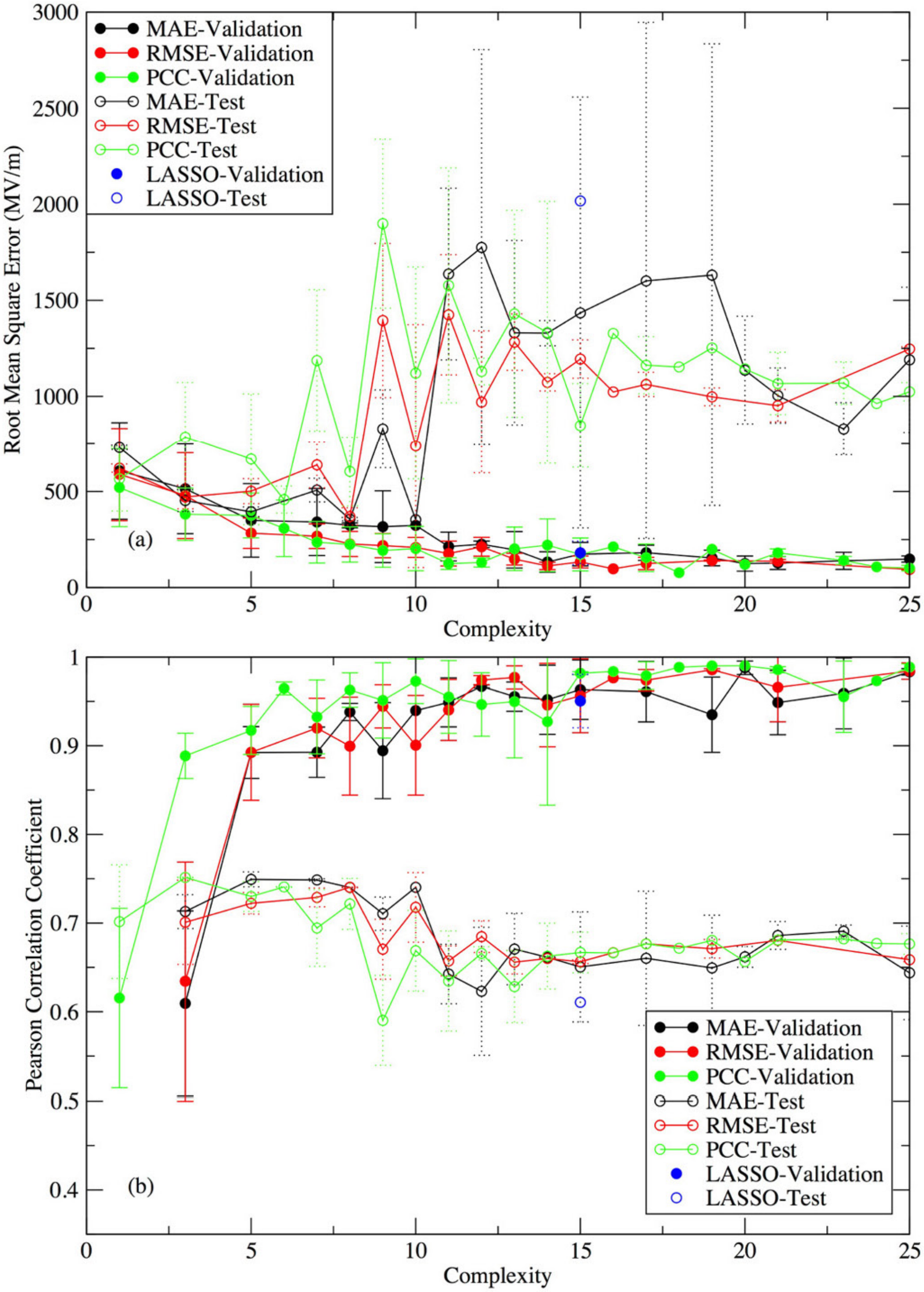

Supplementary Figure S7. (a) RMSE and (b) PCC performance on total input data validation data and test data after MAE, RMSE, PCC optimizations. The LASSO solution is also shown. The error bar is calculated using the standard deviation from 16 parallel runs. The output value for these runs was the dielectric breakdown strength.

11. Universal Pareto frontiers including test data

We constructed a universal Pareto frontier based on model performance on all data (i.e., training+validation+test data) for models discovered using the training and validation data, with the logarithm of the dielectric breakdown strength as the output value. All models in this universal Pareto frontier are tabulated in Supplementary Table S7 and are plotted against the complexity in Supplementary Fig. S8. These are comparable to Table 2 and Fig. 8 in the main text.

No model with complexity above 28 appears on this Pareto frontier (Supplementary Fig. S8). The model quality improves quickly up to complexity 9. Afterwards, the improvement slows down dramatically. For complexity ranging from 9 to 13, models on Pareto frontier show a similar predictive accuracy (Supplementary Fig. S8) as well as a similar tree structure (Supplementary Table S7).

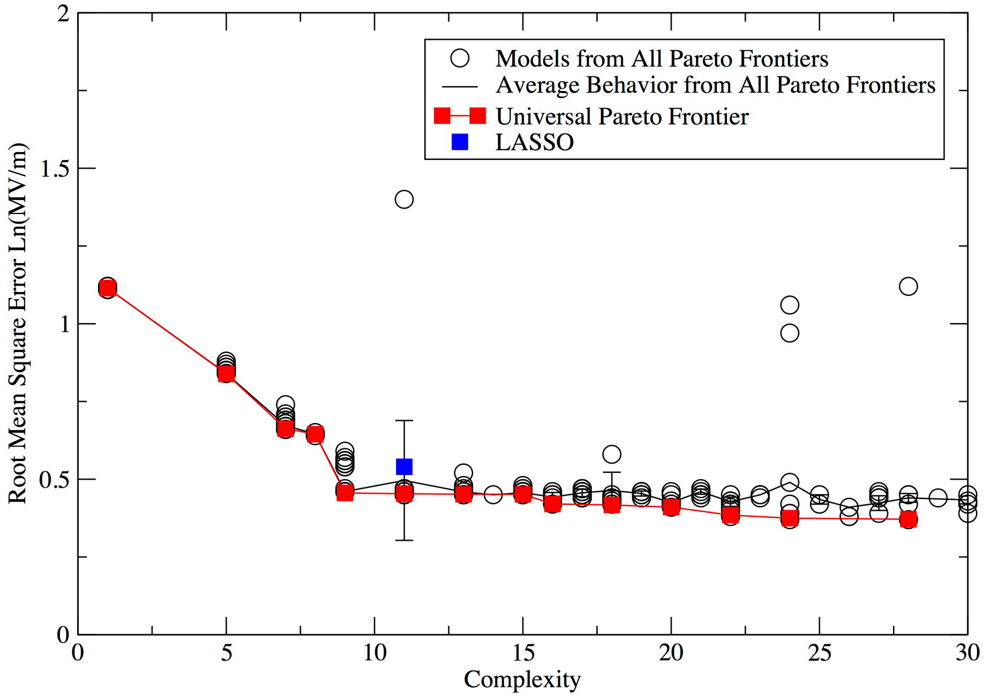

Supplementary Figure S8. RMSE performance of models on all data (i.e., training+validation+test) for LASSO, the universal Pareto frontier, and models from all Pareto frontiers trained on all data when using the natural logarithm of the dielectric breakdown strength as the output value.

Complexity	Model	Benchmark	ε_{al}	ε_{tr}	ε_{va}
1	5.33	RMSE	1.11	1.12±0.09	1.11±0.08
		PCC	N/A	N/A	N/A
5	$4.32 + 0.0805\omega_{\max}$	RMSE	0.84	0.82±0.07	0.85±0.07
		PCC	0.66	0.68±0.05	0.64±0.05
7	$4.5 + 0.0139E_g\omega_{\max}$	RMSE	0.66	0.66±0.07	0.66±0.07
		PCC	0.80	0.81±0.03	0.81±0.03
8	$7.25 - 25.6/(E_g + \omega_{\max})$	RMSE	0.64	0.61±0.07	0.67±0.06
		PCC	0.82	0.84±0.04	0.80±0.05
9	$1.73 + \ln(\omega_{\max}) + \ln(E_g)$	RMSE	0.46	0.44±0.04	0.47±0.04
		PCC	0.91	0.92±0.02	0.90±0.02
11	$1.7 + \ln(\omega_{\max}) + \ln(E_g + 0.11)$	RMSE	0.45	0.43±0.04	0.47±0.04
		PCC	0.91	0.92±0.02	0.91±0.02
13	$1.66 + \ln(\omega_{\max}) + \ln(E_g + 0.0182\omega_{\max})$	RMSE	0.45	0.43±0.04	0.47±0.04
		PCC	0.91	0.92±0.02	0.91±0.02
15	$1.05\ln(\omega_{\max} + 5.5E_g\omega_{\max}) - 0.311$	RMSE	0.45	0.43±0.04	0.47±0.04
		PCC	0.91	0.92±0.02	0.91±0.02
16	$\ln(5.82E_g\omega_{\max} + \frac{E_g}{E_g - 2.11})$	RMSE	0.42	0.41±0.04	0.42±0.03
		PCC	0.93	0.93±0.01	0.92±0.01

18	$1.025\ln(E_g\omega_{\max}+\frac{E_g}{\omega_{\max}-19.5})$ $+1.67$	RMSE	0.42	0.41±0.04	0.42±0.03
		PCC	0.93	0.93±0.01	0.93±0.01
20	$1.124\ln(2.69+E_g\omega_{\max}$ $+\frac{1.05}{E_g-2.13})+1.18$	RMSE	0.41	0.41±0.03	0.41±0.03
		PCC	0.93	0.93±0.01	0.93±0.01
22	$1.091\ln((0.547+E_g)\omega_{\max}$ $+\frac{13.5}{\omega_{\max}(E_g-2.12)})+1.17$	RMSE	0.38	0.38±0.03	0.39±0.03
		PCC	0.94	0.94±0.01	0.93±0.02
24	$1.191\ln(E_g\omega_{\max}+\omega_{\max}+E_g$ $+\frac{8.32}{(\omega_{\max}-2.46)(E_g-2.11)})$ $+0.713$	RMSE	0.37	0.37±0.03	0.37±0.03
		PCC	0.94	0.94±0.01	0.94±0.01
28	$1.106\ln(2E_g\omega_{\max}+\omega_{\max}$ $+\frac{12.9}{(\omega_{\max}-2.31)(E_g-2.11)})$ $+0.357$	RMSE	0.37	0.37±0.02	0.37±0.03
		PCC	0.94	0.94±0.01	0.94±0.01
11 (LASSO)	$3.1963+0.315\sqrt{E_g\omega_{\max}}$	RMSE	0.54	0.53±0.05	0.54±0.04
		PCC	0.88	0.89±0.02	0.88±0.02

Supplementary Table S7. The performance in predicting the natural logarithm of dielectric breakdown strength on all data ε_{al}, training data ε_{tr}, and validation data ε_{va} for models on the universal Pareto frontier constructed using training, validation, and test data.

12. Performance of four representative models when using the dielectric breakdown strength as the output value

The predicted dielectric breakdown strength of four models S1-S4 is compared between density functional theory and models discovered using machine learning is shown in Supplementary Fig. S9. A zoom-in view where F_b ranges from 0 to 3000 MV/m is plotted in Supplementary Fig. S10. In addition, a log-log scale plot is shown in Supplementary Fig. S11 to give readers a direct sense of magnitude of dielectric breakdown strength.

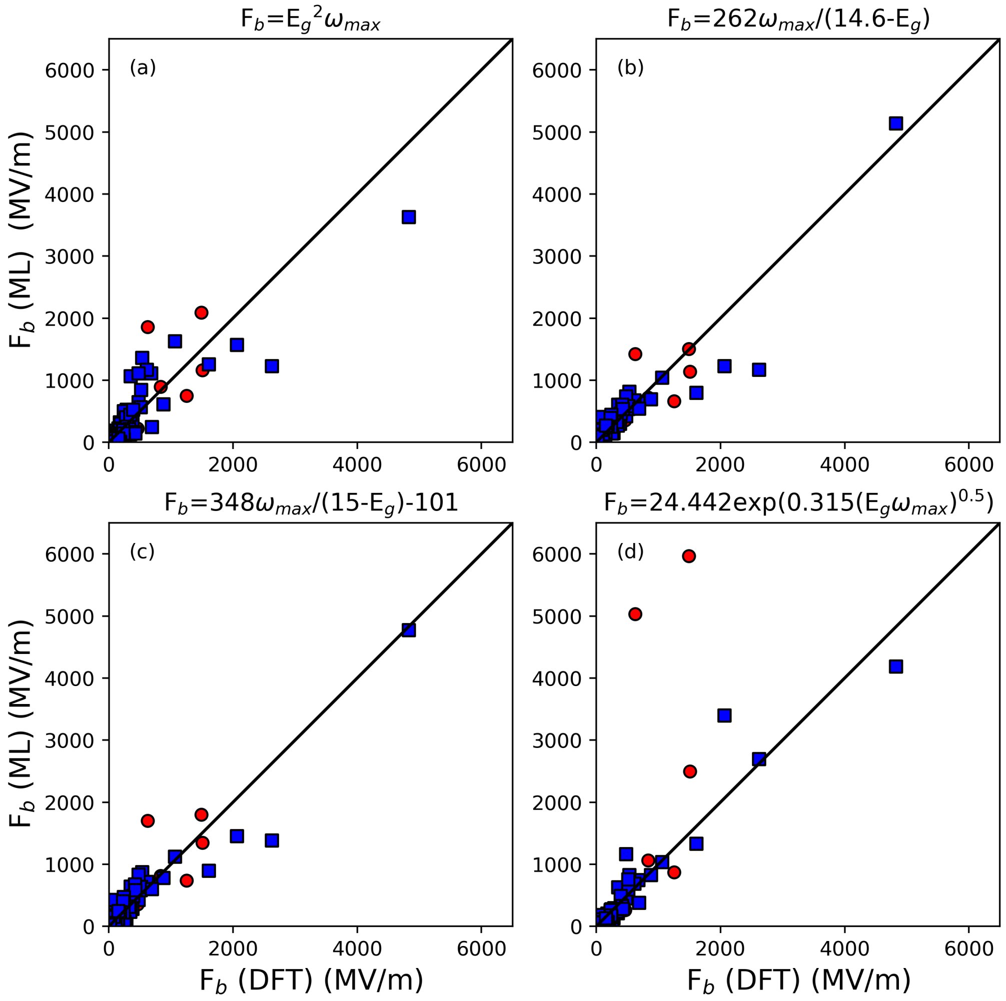

Supplementary Figure S9. Dielectric breakdown strength F_b predicted by machine learning (ML) and density functional theory (DFT) for (a) S1, (b) S2, (c) S3 and (d) S4 (the LASSO solution). Blue squares represent the prediction for all training and validation data and red circles represent the prediction for test data. The black solid line indicates a perfect match between ML and DFT.

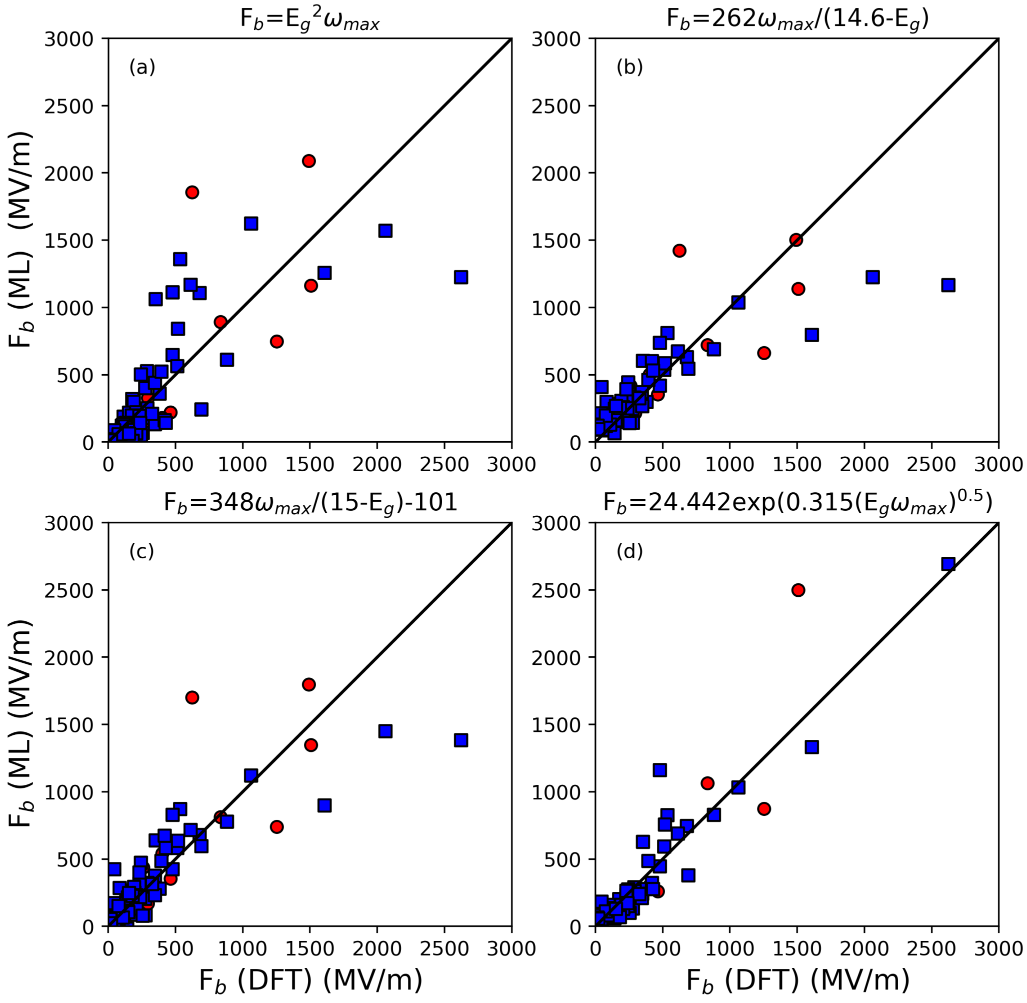

Supplementary Figure S10. Zoom-in plot of dielectric breakdown strength F_b predicted by machine learning (ML) and density functional theory (DFT) for (a) S1, (b) S2, (c) S3 and (d) S4 (the LASSO solution). Blue squares represent the prediction for all training and validation data and red circles represent the prediction for test data. The black solid line indicates a perfect match between ML and DFT.

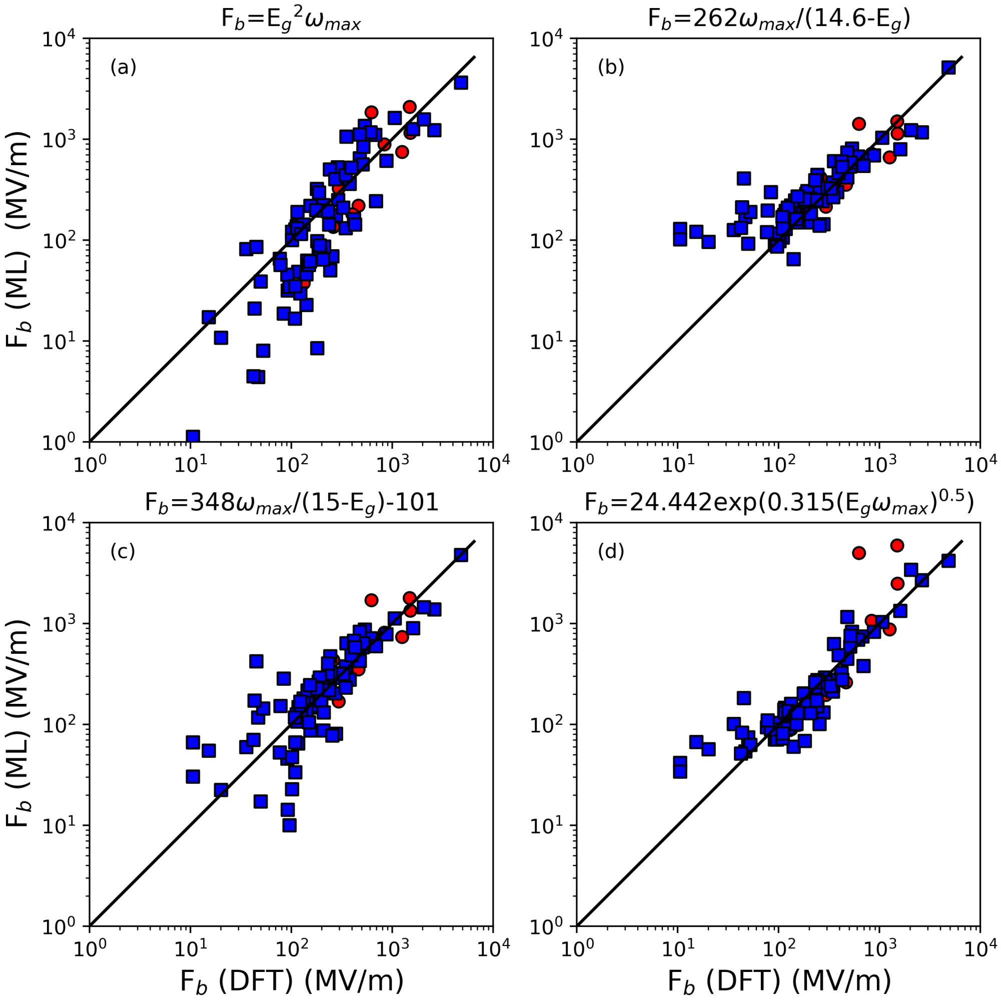

Supplementary Figure S11. Dielectric breakdown strength F_b predicted by machine learning (ML) and density functional theory (DFT) for (a) S1, (b) S2, (c) S3 and (d) S4 (the LASSO solution) on a log-log scale. Blue squares represent the prediction for all training and validation data and red circles represent the prediction for test data. The black solid line indicates a perfect match between ML and DFT.

13. Universal Pareto frontier created by including the test data when determining model performance.

We have constructed a universal Pareto frontier of models discovered using the training and validation data, but where the performance of the models was evaluated against the training+validation+test when constructing the Pareto frontier. The dielectric breakdown strength was used as the output value. This frontier (Supplementary Fig. S12) has no model with complexity above 10, providing additional evidence that more complex models overfit the training data. The best models on this frontier (Supplementary Table S7) have a similar form as models S2 and S3, providing further evidence of the predictive power of models of this form.

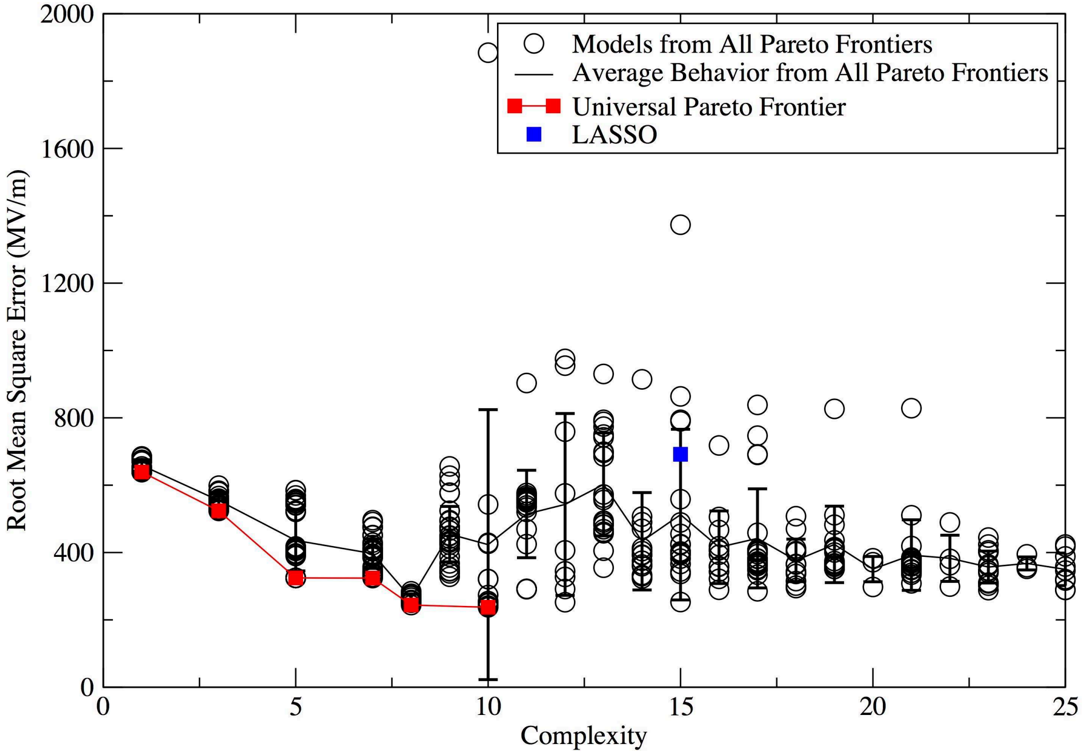

Supplementary Figure S12. Root mean square error (RMSE) performance of models on all data (i.e., training+validation+test) for the LASSO solution, the universal Pareto frontier created by including the test data when calculating the RMSE, and models from all Pareto frontiers. Dielectric breakdown strength is used as the output value.

Complexity	Model	Benchmark	ε_{al}	ε_{tr}	ε_{va}	ε_{tt}
1	387.9	RMSE	644	604±232	580±231	597
		PCC	N/A	N/A	N/A	N/A
3	$35.2\omega_{\max}$	RMSE	524	492±221	476±220	381
		PCC	0.58	0.63±0.10	0.61±0.11	0.73
5 (S1)	$E_g^2\omega_{\max}$	RMSE	324	289±62	289±65	489
		PCC	0.86	0.87±0.07	0.86±0.09	0.72
7	$E_g^2\omega_{\max} - E_g$	RMSE	324	288±63	288±66	487
		PCC	0.86	0.87±0.07	0.86±0.09	0.72
8 (S2)	$\frac{277\omega_{\max}}{14.7 - E_g}$	RMSE	244	220±53	220±51	351
		PCC	0.93	0.92±0.04	0.90±0.05	0.74
10 (S3)	$\frac{340\omega_{\max}}{14.9 - E_g} - 74$	RMSE	237	201±46	206±45	403
		PCC	0.93	0.92±0.05	0.91±0.05	0.74
15 (S4)	$24.442\,\mathrm{e}^{0.315\sqrt{E_g\omega_{\max}}}$	RMSE	692	202±62	174±59	2017
(LASSO)		PCC	0.74	0.95±0.03	0.95±0.03	0.61

Supplementary Table S8. The performance in predicting dielectric breakdown strength on all data ε_{al}, training data ε_{tr}, validation data ε_{va} and test data ε_{tt} for models on the universal Pareto frontier created by including the test data when calculating the RMSE.

References

1 Schmidt, M. & Lipson, H. Distilling Free-Form Natural Laws from Experimental Data. *Science* **324**, 81-85, doi:10.1126/science.1165893 (2009).

2 Kim, C., Pilania, G. & Ramprasad, R. Machine Learning Assisted Predictions of Intrinsic Dielectric Breakdown Strength of ABX3 Perovskites. *J. Phys. Chem. C* **120**, 14575-14580, doi:10.1021/acs.jpcc.6b05068 (2016).

3 Kim, C., Pilania, G. & Ramprasad, R. From Organized High-Throughput Data to Phenomenological Theory using Machine Learning: The Example of Dielectric Breakdown. *Chemistry of Materials*, doi:10.1021/acs.chemmater.5b04109 (2016).

4 Liu, D. C. & Nocedal, J. On the limited memory BFGS method for large scale optimization. *Mathematical Programming* **45**, 503-528, doi:10.1007/BF01589116 (1989).

5 Nocedal, J. Updating quasi-Newton matrices with limited storage. *Math. Comp.* **35**, 773-782, doi:10.1090/S0025-5718-1980-0572855-7 (1980).
